# Genome sequencing provides insights into the evolution and antioxidant activity of Chinese bayberry

**DOI:** 10.1186/s12864-019-5818-7

**Published:** 2019-06-06

**Authors:** Haiying Ren, Haiyan Yu, Shuwen Zhang, Senmiao Liang, Xiliang Zheng, Shujian Zhang, Pu Yao, Hongkun Zheng, Xingjiang Qi

**Affiliations:** 1 Institute of Horticulture, Zhejiang Academy of Agricultural Sciences, Hangzhou, China; 2 Biomarker Technologies Corporation, Beijing, China; 3 Plant Pathology Department, University of Florida, Gainesville, Florida, USA

**Keywords:** Chinese bayberry, genome, transcriptome, evolution, antioxidant activity

## Abstract

**Background:**

Chinese bayberry (*Myrica rubra* Sieb. & Zucc.) is an economically important fruit tree characterized by its juicy fruits rich in antioxidant compounds. Elucidating the genetic basis of the biosynthesis of active antioxidant compounds in bayberry is fundamental for genetic improvement of bayberry and industrial applications of the fruit’s antioxidant components. Here, we report the genome sequence of a multiple disease-resistant bayberry variety, ‘Zaojia’, in China, and the transcriptome dynamics in the course of fruit development.

**Results:**

A 289.92 Mb draft genome was assembled, and 26,325 protein-encoding genes were predicted. Most of the *M. rubra* genes in the antioxidant signaling pathways had multiple copies, likely originating from tandem duplication events. Further, many of the genes found here present structural variations or amino acid changes in the conserved functional residues across species. The expression levels of antioxidant genes were generally higher in the early stages of fruit development, and were correlated with the higher levels of total flavonoids and antioxidant capacity, in comparison with the mature fruit stages. Based on both gene expression and biochemical analyses, five genes, namely, caffeoyl-CoA O-methyltransferase, anthocyanidin 3-O-glucosyltransferase, (+)-neomenthol dehydrogenase, gibberellin 2-oxidase, and squalene monooxygenase, were suggested to regulate the flavonoid, anthocyanin, monoterpenoid, diterpenoid, and sesquiterpenoid/triterpenoid levels, respectively, during fruit development.

**Conclusions:**

This study describes both the complete genome and transcriptome of *M. rubra*. The results provide an important basis for future research on the genetic improvement of *M. rubra* and contribute to the understanding of its genetic evolution. The genome sequences corresponding to representative antioxidant signaling pathways can help revealing useful traits and functional genes.

**Electronic supplementary material:**

The online version of this article (10.1186/s12864-019-5818-7) contains supplementary material, which is available to authorized users.

## Background

The Myricaceae family comprises three genera, of which *Myrica*, comprising approximately 50 species, is most widely distributed across the warm, humid regions of Asia (e.g., China, Japan, and India), Europe (e.g., France, Norway, Switzerland, and Spain), Africa (e.g., Kenya), North America (e.g., USA), and South America (e.g., Brazil) [1–4]. Chinese bayberry (*Myrica rubra* Sieb. & Zucc.) is an economically important fruit tree, and its cultivation area in China is about 334,000 ha with an annual yield of 950,000 t, approximately. In addition to producing flavorful fruit, different organs of Chinese bayberry are used in traditional Chinese medicine. Chinese bayberry extracts contain antioxidants against inflammation, allergies, diabetes, cancer, bacterial infections, and diarrhea, among other health issues [1].

Extracts of *Myrica* spp. contain many different kinds of flavonoids, such as myricetin, anthocyanins and proanthocyanidins, cyanidin-3-glucoside, myricanol, and myricanone [5–9], and phenolic compounds, such as gallic acid, catechin, hydroxybenzoic acid, and coumaric acid. Myricanone induces apoptosis in HepG2 liver cancer cells through the generation of reactive oxygen species, depolarization of the mitochondrial membrane, early release of cytochrome-c, downregulation of heat shock protein 70, and activation of a caspase cascade [10]. It also has anticancer effects on the HeLa (cervical cancer) and PC3 (prostate cancer) cell lines as it triggers caspase activation and suppresses cell proliferation by downregulating NF-κB and signal transducer and activators of transcription 3 signaling cascades [10]. Myricetin exerts selective cytotoxic, pro-apoptotic, and anti-metastatic effects on prostate cancer cells by inhibiting *PIM1* and disrupting the PIM1/CXCR4 interactions [11]. Chinese bayberry leaf flavonoids and proanthocyanidins inhibit the growth of the ovarian cancer cell line A2780/CP70. The extract was found to increase the expression of cleaved caspases 3 and -7 and to induce apoptosis via an extracellular signal-regulated kinase-dependent caspase-9 activation intrinsic apoptotic pathway by upregulating the pro-apoptotic proteins (BAD and BAX) and downregulating the anti-apoptotic proteins (BCL-XL and BCL-2) [12]. It also inhibits angiogenesis and induces G1 cell cycle arrest, further reducing the levels of reactive oxygen species and targeting the AKT/mTOR/P70S6K/4E-BP-1 pathway to reduce the expression of hypoxia-inducible factor 1-alpha and vascular endothelial growth factor, thus inhibiting angiogenesis [12, 13]. Treatment of human gastric cancer cells SGC-7901 with cyanidin-3-glucoside markedly increased *KLF6* expression (an important tumor suppressor gene inactivated in many human cancers) and P21 protein levels, and inhibited cyclin-dependent kinase 4 and cyclin D1 expression [7].

Flavonoids, including cyanidin-3-glucoside and anthocyanidins, may have beneficial effects on diabetes by reducing oxidative stress in muscle and fat [14]. Extracts from *M. rubra* showed protective effects on liver injuries [15, 16] and non-alcoholic and alcohol-induced fatty liver disease [17, 18], which may be due to its potent antioxidant properties. Thus, the major flavonoids, proanthocyanidins, and anthocyanins of Chinese bayberry leaves have strong chemical and cellular antioxidant activities [6, 19, 20].

Flavonoids are abundant in Chinese bayberry fruits, but the genetic basis of their biosynthesis remains unclear. Two genes encoding key proanthocyanidins and biosynthetic enzymes, namely anthocyanidin reductase and leucoanthocyanidin reductase, have been isolated from Chinese bayberry fruit cDNA [21]. Partial cDNA sequences of the genes encoding the anthocyanin biosynthesis enzymes chalcone synthase, chalcone isomerase, flavanone 3-hydroxylase (F3H), flavonoid 3'-hydroxylase (F3'H), dihydroflavonol 4-reductase (DFR), anthocyanidin synthase (ANS), and uridine diphosphate glucose flavonoid 3-O-glucosyltransferase (UFGT), as well as *MrMYB1*, a R2R3 MYB transcription factor homologous to known anthocyanin biosynthesis activators, were isolated from ripe Chines bayberry ‘Biqizhong’ fruit. Differences in the mRNA abundance of *MrF3H*, *MrF3'H*, *MrDFR1*, *MrANS*, and *MrUFGT* as well as the transcript level of *MrMYB1*, were highly correlated with the differential accumulation of anthocyanins [22]. The sequences, active sites, and expression levels of the genes related to the antioxidant signaling pathway in Chinese bayberry remain unknown, but elucidating them would be useful for the development of new medication.

Recently, Jia et al. reported the genome sequence of a female ‘Shuijing’ Chinese bayberry individual bearing near-white fruit producing little anthocyanin [23]. The genome size was 313 Mb, 90 % of sequences were assembled into eight pseudo-chromosomes, 32,493 genes were predicted, and a 59-kb genomic region presumably related to sex determination in the female was identified [23]. In the present study we sequenced and assembled the genome of a different Chinese bayberry cultivar, ‘Zaojia’, bearing anthocyanins-rich fruit. We also conducted in-depth transcriptomic analyses to reveal the gene regulatory basis underlying its antioxidant and pharmacological activity.

## Results and discussion

### Genome assembly and annotation

We adopted a whole-genome shotgun strategy to sequence and assemble the genome, yielding a total of 64,147 Mb at a depth (×) of 210.75 (Additional file 1: Table S1). The assembled genome was 289.92 Mb, which was similar to the size of the assembled ‘Shuijing’ genome and covered 95.05 % of the estimated genome of ‘Zaojia’ (304.38 Mb) (Additional file 1: Table S2; Additional file 2: Figure S1a) [23]. However, it was much smaller than the genomes of apple (603.9 Mb), grapevine (487 Mb), and orange (320.5 Mb), but larger than that of peach (227.4 Mb) (Additional file 1: Table S3). We predicted 26,325 protein-encoding genes with an average gene length of 3,828 bp, exon length of 266 bp, and intron length of 488.17 bp (Additional file 1: Table S4; Additional file 2: Figure S2).

### Whole-genome duplication in Chinese bayberry

**Fig. 1:**
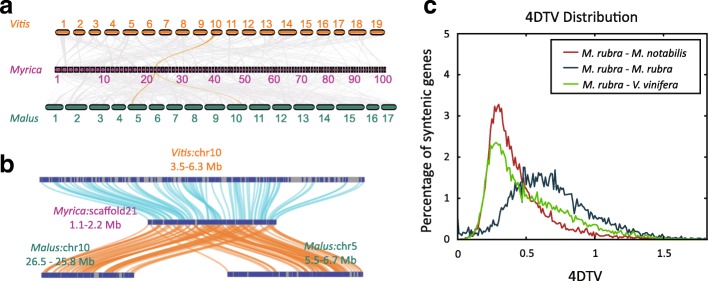
Whole-genome duplication (WGD) in Chinese bayberry, *Myrica rubra*. (a) Macrosynteny of *Vitis vinifera*, *Myrica rubra*, and *Malus × domestica*. Genomic alignment of *V. vinifera*, *M. rubra*, and *M. × domestica* shows that one ancestral region in the basal eudicot *V. vinifera* can be aligned to one region in *M. rubra* derived from the paleohexaploidization common to eudicots. One region in *M. rubra* can be aligned to two regions in *M. × domestica*, indicating that WGD recently occurred in *M. × domestica*. (**b**) Microcollinearity analysis of *V. vinifera*, *M. rubra*, and *M. × domestica*. These regions represent one collinear set, highlighted in orange in (**a**). Navy blue rectangles indicate the predicted genes in the genome. One gene in *V. vinifera* corresponds to one gene in *M. rubra*, whereas one gene in *M. rubra* has two homologs in *M.* × *domestica*. (**c**) Density distribution of fourfold synonymous third-codon transversions (4DTV) for paralogous pairs among *M. rubra, M. notabilis*, and *V. vinifera*. The single peak of the *M. rubra–M. rubra* paralogs indicates no recent WGD in *M. rubra* except for the paleohexaploidization event, which is common to eudicots.

Within the Chinese bayberry genome, 14,303 paralogous relationships were identified, covering 31 % of the genes (Additional file 1: Table S5). Genomic synteny among *M. rubra*, *Vitis vinifera* (grapevine), and *Malus* × *domestica* (apple) revealed the genomic evolution of *M. rubra* (Figure 1a and b). An ancestral genome region in the basal eudicot *V. vinifera* was aligned to a genome region of *M. rubra* indicating paleohexaploidization, which is common in eudicots, but no recent whole-genome duplication event was detected in *M. rubra* (Figure 1a and b). One genome region of *M. rubra* aligned with two regions of *Malus × domestica*, indicating a recent whole-genome duplication event in the latter (Figure 1 a and 1b). A comparison of the Chinese bayberry genome sequence with that of *Morus notabilis* (mulberry) and *V. vinifera* revealed a large number of syntenic regions (Figure 1c). The distribution of fourfold synonymous third-codon transversions (Figure 1c) and synonymous nucleotide substitution (Ks) rates (Additional file 2: Figure S3) of homologous pairs in the syntenic regions, as well as the mean Ks values of individual syntenic regions of individual syntenic blocks, indicated that only the paleohexaploidization events common to eudicots occurred in the evolutionary history of Chinese bayberry. The whole-genome duplication event occurred before the *M. rubra*–*M. notabilis* or *M. rubra*–*V. vinifera* divergence and it is shared among eudicots.

### Comparative genomic analysis

**Fig. 2:**
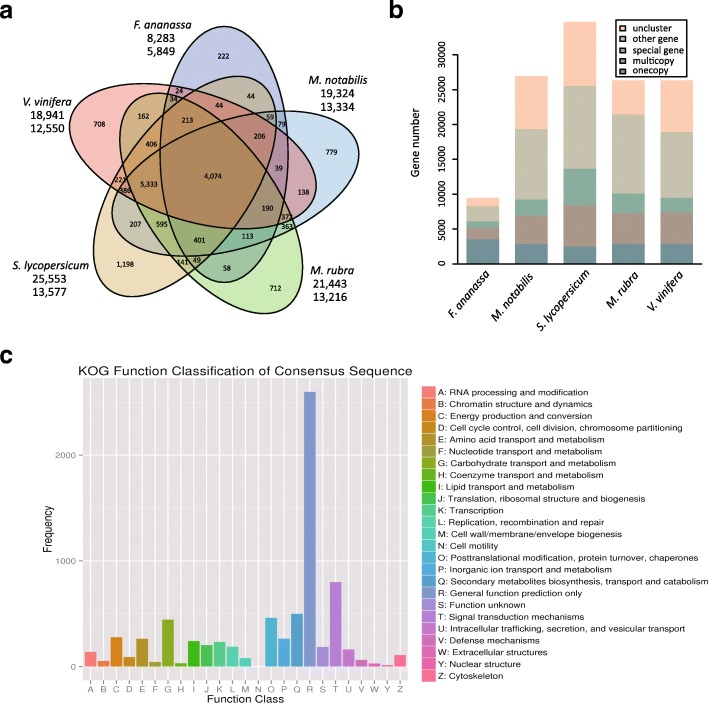
Comparative genomic analysis of *Myrica rubra*, *Solanum lycopersicum*, *Morus notabilis*, *Fragaria × ananassa*, and *Vitis vinifera*. (**a**) Clustering of gene families by OrthoMCL in *M. rubra*, *S. lycopersicum*, *M. notabilis*, *F. × ananassa*, and *V. vinifera*. (**b**) Statistics of the genes involved in clustering. (**c**) Annotation and classification of genes unique to *M. rubra* by KOG.

Gene similarity clustering of the 26,325 predicted genes for *M. rubra* with those of *Solanum lycopersicum*, *V. vinifera*, *Fragaria* × *ananassa*, and *M. notabilis* yielded 13,216 unique gene families (Additional file 1: Table S6). A five-way comparison showed that 4,074 gene families were shared by the five species, and 712 gene families were unique to *M. rubra*. This number was higher than in *F.* × *ananassa* (222), lower than in *S. lycopersicum* (1,198), and similar to that in *M. notabilis* (779) and *V. vinifera* (708) (Figure 2a and b). Gene similarity clustering of the 26,325 predicted genes for *M. rubra* with those of *Cicer arietinum*, *Arabidopsis thaliana*, and *Oryza sativa* yielded 12,266 unique gene families (Additional file 1: Table S7). A four-way comparison revealed that 4,270 gene families were shared by these four species, and 891 gene families were unique to *M. rubra*. This number was higher than that in *C. arietinum* (214) and lower than that in *A. thaliana* (933) and *O. sativa* (2,478) (Additional file 2: Figure S4a and S4b).

The classification of specific genes in the above two classes (the above five-way comparison and the four-way comparison) was similar in different gene families. The first four groups were involved in signal transduction mechanisms (800, 858), secondary metabolite biosynthesis, transport, and catabolism (500, 509), post-translational modification, protein turnover, and chaperones (461, 535), and carbohydrate transport and metabolism (445, 458) (Figure 2c; Additional file 2: Figure S4c). This indicates that these functions were enhanced during Chinese bayberry evolution. The antioxidant signaling pathways of flavonoids, anthocyanins, and terpenes in *M. rubra* comprise genes of the abovementioned four groups, and it is suggested that they are enhanced in *M. rubra* in comparison with other plant species.

A phylogenetic tree based on 560 single-copy protein sequences was constructed for nine species (*M. rubra*, *S. lycopersicum*, *Prunus mume*, *M. notabilis*, *Citrullus lanatus*, *F.* × *ananassa*, *Citrus sinensis*, *A. thaliana,* and *Carica papaya*) using PhyML. *Myrica rubra*, *P. mume*, and *M. notabilis* were clustered in a subclade that was most likely derived from a common ancestor approximately 93.95 Mya, whereas *P. mume* and *M. notabilis* diverged 82.41 Mya, and *C. lanatus* diverged from an ancestor shared with *M. rubra*, *P. mume*, and *M. notabilis* around 97.67 Mya. The two clades, one including *M. rubra*, *C. lanatus*, *P. mume*, and *M. notabilis* and the other including *C. sinensis*, *F.* × *ananassa*, *A. thaliana*, and *C. papaya*, were estimated to have diverged 103.30 Mya (Additional file 2: Figure S5a).

A phylogenetic tree inferred from 1,737 single-copy protein sequences of six species (*M. rubra*, *S. lycopersicum*, *P. mume*, *M. notabilis*, *C. lanatus*, and *F.* × *ananassa*) resolved *M. rubra* as sister to the clade of *P. mume* and *M. notabilis* (Additional file 2: Figure S5b). Comparison of the gene families in *S. lycopersicum*, *P. mume*, *M. notabilis*, *C. lanatus*, and *F.* × *ananassa* with those in *M. rubra* led to the identification of 513 gene families that were expanded (Additional file 1: Table S8), such as leucine-rich repeats (six copies), toxic anion resistance protein, and reverse transcriptase, and 4,063 gene families that were contracted in *M. rubra* (Additional file 1: Table S9). Moreover, the MYB/SANT-like DNA-binding domain, zinc-binding domain in reverse transcriptase, transposase family tnp2, and transposase-associated domain exhibited more significant gene family expansion when compared with those of other plants (Additional file 1: Table S8), indicating that these functions have been enhanced during Chinese bayberry evolution.

### Gene analysis of antioxidant and pharmacological activity

**Fig. 3:**
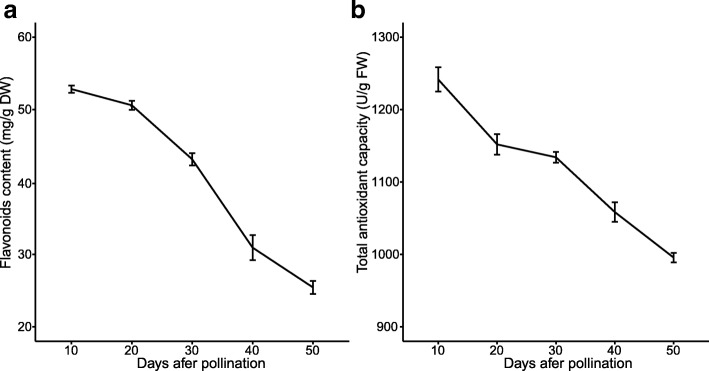
Changes in (**a**) total flavonoid content and (**b**) total antioxidant capacity during the growth and development of *Myrica rubra* fruit.

**Fig. 4:**
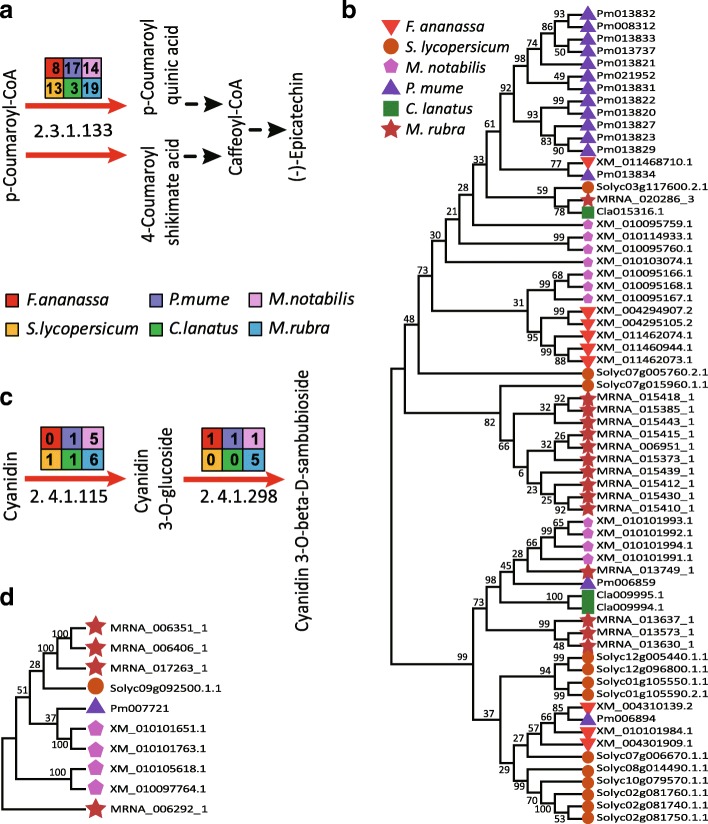
Gene expansions in flavonoid and anthocyanin biosynthesis pathways. Flavonoid (**a**) and anthocyanin (**c**) biosynthesis in plants. Enzyme Commission number (EC) 2.3.1.133, shikimate *O*-hydroxycinnamoyltransferase (HCT). EC 2.4.1.115, anthocyanidin 3-*O*-glucosyltransferase; EC 2.4.1.298, anthocyanidin 3-*O*-glucoside 5-*O*-glucosyltransferase (UGT75C1). Phylogenetic analysis of shikimate *O*-HCT (**b**) and UGT75C1 (**d**) in *Solanum lycopersicum*, *Prunus mume*, *Morus notabilis*, *Myrica rubra*, *Citrullus lanatus*, and *Fragaria* × *ananassa*. Values at the nodes indicate the percentage of bootstrap support from 1,000 replicates

**Fig. 5:**
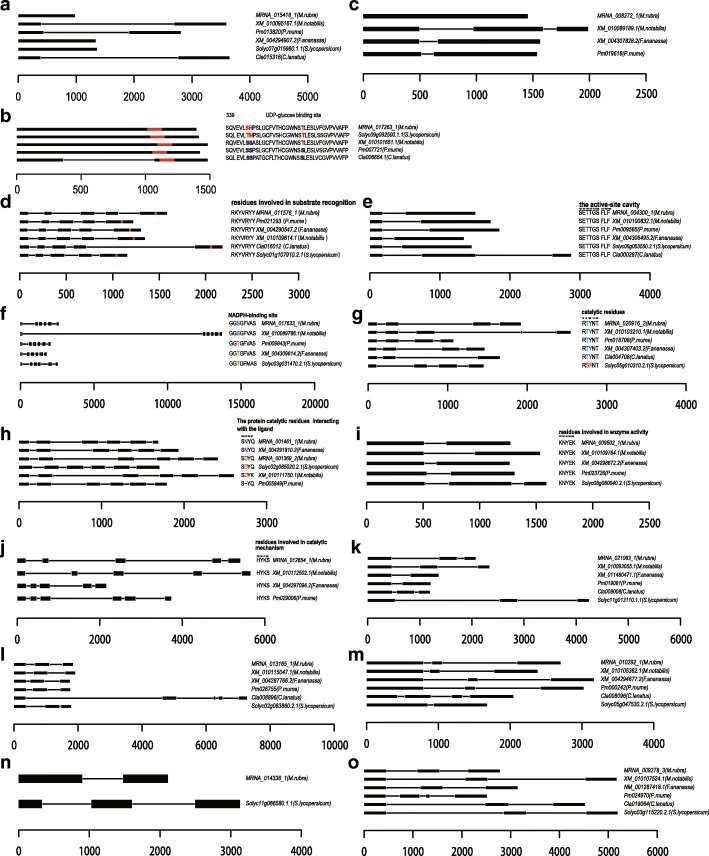
Gene structure and/or single amino acid changes in functionally active residues for gene expansions in flavonoid and anthocyanin biosynthesis pathways. (**a**) shikimate O-hydroxycinnamoyltransferase (HCT, K13065). (**b**) anthocyanidin 3-O-glucoside 5-O-glucosyltransferase (UGT75C1, K12338). (**c**) anthocyanidin 3-O-glucosyltransferase (BZ1, K12930). (**d**) Caffeoyl-CoA O-methyltransferase (CCoAOMT, K00588). (**e**) chalcone synthase (CHS, K00660). (**f**) anthocyanidin reductase (ANR, K08695). (**g**) chalcone isomerase (CHI, K01859). (**h**) bifunctional dihydroflavonol 4-reductase/flavanone 4-reductase (DFR, K13082). (**i**) leucoanthocyanidin dioxygenase (LDOX, K05277). (**j**) leucoanthocyanidin reductase (LAR, K13081). (**k**) flavonol synthase (FLS, K05278). (**l**) naringenin 3-dioxygenase (K00475). (**m**) trans-cinnamate 4-monooxygenase (CYP73A, K00487). (**n**) flavonoid 3',5'-hydroxylase (K13083). (**o**) flavonoid 3'-monooxygenase (K05280)

**Fig. 6:**
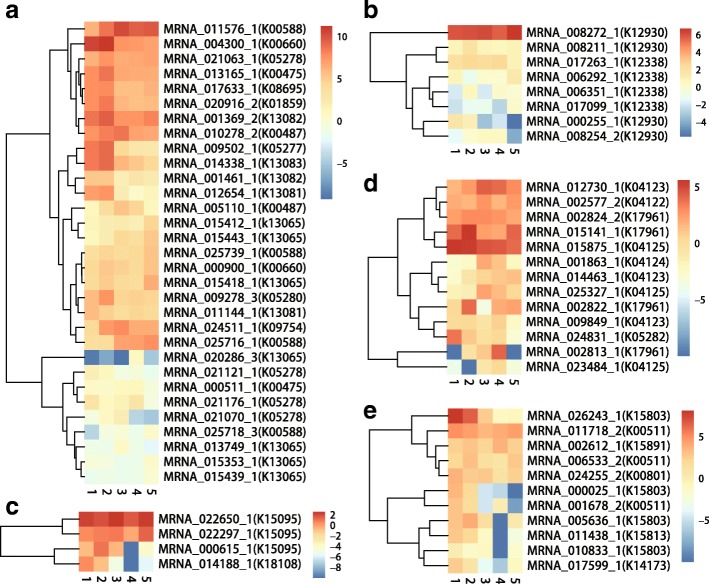
Expression profiles of functional genes involved in (**a**) flavonoid, (**b**) anthocyanin, (**c**) monoterpenoid, (**d**) diterpenoid, and (**e**) sesquiterpenoid and triterpenoid biosynthesis pathways in *Myrica rubra.* Numbers 1, 2, 3, 4, and 5 denote 10, 20, 30,40, and 50 days after pollination, respectively.

11The antioxidant activity of Chinese bayberry extract and its ability to inhibit cancer cell reproduction are linked to total flavonoids, anthocyanins, and terpenes [24, 25]. We found that the fruit of ‘Zaojia’ have relatively high total flavonoid contents, and that their antioxidant activity decreased during the course of development (Figure 3; Additional file 1: Table S10). According to the Kyoto Encyclopedia of Genes and Genomes (KEGG) signaling pathway for flavonoid biosynthesis (reference map 00941), the copy number of the enzyme-coding genes within the flavonoid biosynthetic pathway in *M. rubra*, *C. lanatus*, *F.* × *ananassa*, *M. notabilis*, *P. mume*, and *S. lycopersicum* were analyzed and compared (Additional file 1: Table S11). In the flavonoid pathway of Chinese bayberry, shikimate *O*-hydroxycinnamoyltransferase (*HCT*) [26] was present in high copy numbers (19) (Figure 4a; Additional file 1: Table S11). Its main role is transferring acetyl groups from *p*-coumaroyl-CoA and caffeoyl CoA to shikimate and quinate [25, 27]. In the phylogenetic tree based on the *HCT* gene families of the six plant species, most of the genes encoding HCT in *M. rubra* were clustered in one subclade (Figure 4b), suggesting a tandem duplication event in this species. The gene structure and/or single amino acid change in functionally active residues of *HCT* (MRNA_014518) showed low similarity to genes of the other five plant species (Figure 5a). The expression levels of *HCT* (K13065) homologs during fruit development were generally low, and the expression levels of ten and nine HCT family members were negatively correlated with total flavonoids and total antioxidant capacity, respectively (Figure 6a; Additional file 1: Table S12). Thus, the high gene copy number and the specific active residues of each gene copy might not be correlated with high contents of flavonoids in Chinese bayberry fruit (Additional file 1: Table S10; Additional file 2: Figure S6a and 6b).

**Fig. 7:**
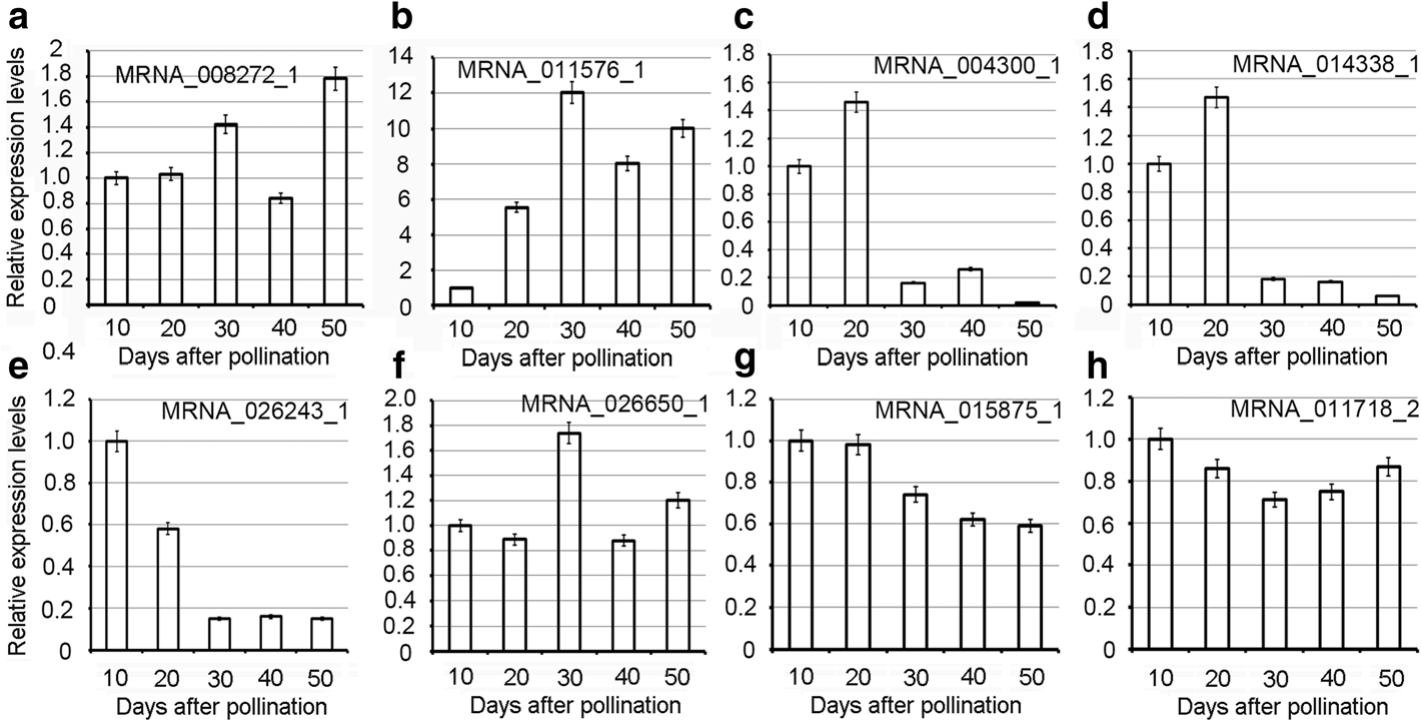
Relative expression levels of eight genes. (**a**) MRNA-008272-1 (anthocyanidin 3-O-glucosyltransferase, BZ1, K12930). (**b**) MRNA-011576-1 (caffeoyl-CoA O-methyltransferase, K005888). (**c**) MRNA_004300_1 (chalcone synthase, K00660). (**d**) MRNA_014338_1 (flavonoid 3',5'-hydroxylase, K13083). (**e**) MRNA_026243_1 ((-)-germacrene D synthase, K15803). (**f**) MRNA-022650-1 ((+)-neomenthol dehydrogenase, K15095). (**g**) MRNA_015875_1 (gibberellin 2-oxidase, K04125). (**h**) MRNA_011718_2 (squalene monooxygenase, K00511)

In the KEGG reference map 00942 for the anthocyanin synthesis signaling pathway, the copy number of anthocyanidin 3-*O*-glucoside 5-*O*-glucosyltransferase (*UGT75C1*; six copies) [28] and anthocyanidin 3-*O*-glucosyltransferase (*BZ1*; five copies) [29] was high (Figure 4c; Additional file 1: Table S11). These two enzymes play important roles in anthocyanin synthesis [1, 30, 31]. The phylogenetic tree based on UGT75C1 confirmed tandem duplication in some genes (Figure 4d). Phylogenetic analysis of the UGT75C1 gene families resolved the homologous genes from *S. lycopersicum* that are closely related to the gene cluster of *M. rubra*. Variations in the functional residues of UGT75C1 (MRNA_017263) compared with its orthologs in *S. lycopersicum*, *M. notabilis*, *P. mume*, and *F.* × *ananassa* (Figure 5b), and *BZ1* (MRNA_008272_1) also showed low similarity across species (Figure 5c). Although UGT75C1 (K12338) had six copies, low expression levels were detected and the expression levels of four copies were negatively correlated with the decrease of total flavonoids and total antioxidant capacity during fruit development (Figure 6b; Additional file 1: Table S13). Because BZ1 (K12930, MRNA_008272_1) is highly expressed throughout the ripening process, it may contribute to the accumulation of anthocyanin during fruit ripening (Figure 6b; Figure 7a; Additional file 1: Table S13). The other four *BZ1* copies detected were positively correlated with the decrease of total flavonoids and total antioxidant capacity during fruit development (Additional file 1: Table S13). Thus, it is reasonable to argue that the high copy number and the variations on the functional residues of the *BZ1* gene (MRNA_008272_1) might have contributed to the high anthocyanin content in ‘Zaojia’ fruit (Additional file 1: Table S10; Additional file 2: Figure S6a and 6b).

The expression patterns of genes related to flavonoid biosynthesis indicated that the pathway is active in *M. rubra* during fruit ripening (Figure 6a and 6b; Additional file 1: Table S12 and S13). Caffeoyl-CoA O-methyltransferase (CCoAOMT) (K005888, MRNA-011576-1), which is associated with the phenylpropanoid pathway and lignin production, showed high expression levels during fruit ripening (Figure 6a; Figure 7b; Additional file 1: Table S12). Its functional residues are highly similar to those in the other five species (Figure 5d). Based on these results, we propose that the high expression levels of CCoAOMT may have contributed to the high flavonoid content in ‘Zaojia’ fruit (Additional file 1: Table S10; Additional file 2: Figure S6a). In maize (*Zea mays*), *ZmCCoAOMT2* confers resistance to both southern leaf blight and gray leaf spot [32]. In *V. vinifera*, VvCCoAOMT is a multifunctional O-methyltransferase that may contribute to anthocyanin methylation activity in grape berries, particularly under drought stress conditions [32, 33]. Resistance in ‘Zaojia’ fruit might be due to variations in *CCoAOMT* (MRNA-011576-1) gene expression levels, which result in differences in the levels of lignin and other metabolites of the phenylpropanoid pathway and in the regulation of programmed cell death. Chalcone synthase is the key enzyme of flavonoid/isoflavonoid biosynthetic pathways, and it functions in the initial step of flavonoid biosynthesis [34]. Chalcone synthase functional residues of *M. rubra* have high similarity to those in the other five species (Figure 5e). In *M. rubra*, chalcone synthase (K00660, MRNA_004300_1) was highly expressed during the early stage of fruit development (Figure 6a; Figure 7c), suggesting that this gene is important for the high total flavonoid content and total antioxidant capacity of young fruit (Additional file 1: Table S10; Figure 3a and 3b). The attenuated expression of the gene in later fruit development stages was correlated with the decrease of total flavonoids (r = 0.799) and total antioxidant capacity (r = 0.659) (Additional file 1: Table S12). Enzymes K05278 (flavonol synthase, MRNA_021063_1), K00475 (naringenin 3-dioxygenase, MRNA_013165_1), K01859 (chalcone isomerase, MRNA_020916_2), K05277 (leucoanthocyanidin dioxygenase, MRNA_009502_1), K05280 (flavonoid 3'-monooxygenase, MRNA_009278_3), K13081 (leucoanthocyanidin reductase, MRNA_012654_1), K13082 (bifunctional DFR/flavanone 4-reductase, MRNA_001369_2 and MRNA_001461_1), K00487 (trans-cinnamate 4-monooxygenase, MRNA_010278_2, gene *CYP73A*), K13083 (flavonoid 3',5'-hydroxylase, MRNA_014338_1), and K08695 (anthocyanidin reductase, MRNA_017633_1) are crucial for flavonoid biosynthesis [35]. They all exhibited high expression levels in early fruit development, and their decreased expressions were correlated with the decrease of total flavonoids and total antioxidant capacity during fruit maturation (Figure 3a and 3b; Additional file 1: Table S12). These genes are suggested to be important for the high early total flavonoid content in fruit development. The functional residues of anthocyanidin reductase (ANR, K08695) (Figure 5f), chalcone isomerase (CHI, K01859) (Figure 5g), bifunctional dihydroflavonol 4-reductase/flavanone 4-reductase (DFR, K13082) (Figure 5h), leucoanthocyanidin dioxygenase (LDOX, K05277) (Figure 5i), and leucoanthocyanidin reductase (LAR, K13081) (Figure 5j) were relatively similar to that of homologous genes in the other five species. The functional residues of flavonol synthase (K0578, Figure 5k), naringenin 3-dioxygenase (K00475, Figure 5l), CYP73A (K00487, Figure 5m), flavonoid 3’5’-hydroxylase (K13083, Figure 5n), and flavonoid 3’-monooxygenase (K05280, Figure 5o) showed low similarity to homologous genes in the other five species. The leucoanthocyanidin dioxygenase of *Reaumuria trigyna* (Enzyme Commission number 1.14.11.19) is a multifunctional dioxygenase in flavonoid biosynthesis that is involved in enhancing plant responses to NaCl stress [19].

Different alleles encoding leucoanthocyanidin reductase influence resistance against the fungus *Heterobasidion parviporum* in *Picea abies* [36]. The expression profiling of flavonoid hydroxylase genes is influenced by diverse abiotic stresses, including cold, salinity, drought, UV-B radiation, and plant hormone abscisic acid or jasmonic acid treatments [18]. The overexpression of *RrANR* in *Rosa rugosa* results in increased plant tolerance to oxidative stress via increased scavenging of reactive oxygen species and modulation of the abscisic acid signaling pathway [37].

We suggest that K005888 (MRNA-011576-1), which encodes CCoAOMT is the most important enzyme related to flavonoid biosynthesis, while K12930 (BZ1, MRNA_008272_1) is the key enzyme related to anthocyanin biosynthesis during fruit development. Stress resistance in Chinese bayberry fruits might be due to variations in the expression levels of these genes and corresponding amino acid sequences, resulting in differences in the levels of flavonoids and other metabolites of the phenylpropanoid pathway and in the regulation of programmed cell death.

**Fig. 8:**
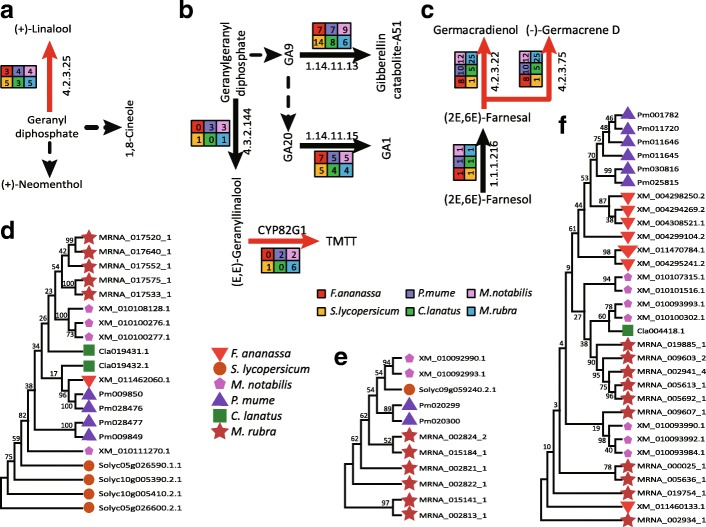
Gene expansions in monoterpenoid, diterpenoid, and sesquiterpenoid/triterpenoid biosynthesis pathways. (**a**) Monoterpenoid biosynthesis pathways in plants. Enzyme Commission number (EC) 4.2.3.25, (3*S*)-linalool synthase. **(b)** Diterpenoid biosynthesis in plants. EC 1.14.11.15, gibberellin 3-beta-dioxygenase; EC 1.14.11.13, gibberellin 2-oxidase; CYP82G1, cytochrome P450, family 82, subfamily G, polypeptide 1; EC 4.2.3.144, geranyllinalool synthase. (**c**) Sesquiterpenoid/triterpenoid biosynthesis. EC 4.2.3.22, 4.2.3.75, (-)-germacrene D synthase; EC 1.1.1.216, farnesol dehydrogenase. Phylogenetic analysis of (3*S*)-linalool synthase family **(d)**, CYP82G1 (**e**), and (-)-germacrene D synthase family (**f**) in *Solanum lycopersicum*, *Prunus mume*, *Morus notabilis*, *Myrica rubra*, *Citrullus lanatus*, and *Fragaria* × *ananassa*. Values at the nodes indicate the percentage of bootstrap support from 1,000 replicates

Regarding the terpenoid signaling pathway, the copy numbers of (3*S*)-linalool synthase (5) [31] of the monoterpene pathway (Fig. 8a; Additional file 1: Table S11), cytochrome P450, 82 family, subfamily g, polypeptide 1 (CYP82G1) (6) of the diterpenes signaling pathway [38] (Fig. 8b; Additional file 1: Table S11), and (-)-germacrene D synthase (K15803) (25) of the sesquiterpene and triterpene synthesis pathway [39] (Fig. 8c; Additional file 1: Table S11) were very high in Chinese bayberry. The phylogenetic trees based on (3*S*)-linalool synthase (Fig. 8d), CYP82G1 (Fig. 8e), and (-)-germacrene D synthase (Fig. 8f) showed a tandem duplication in *M. rubra*.

**Fig. 9:**
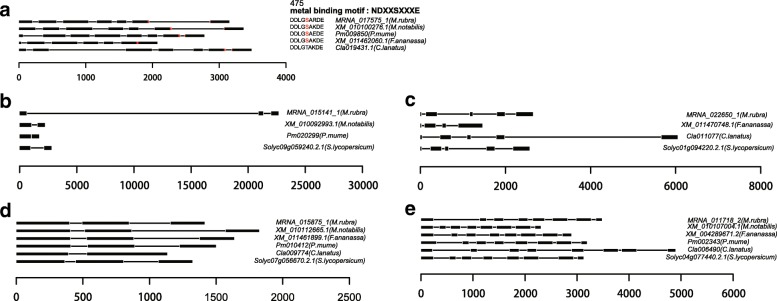
Gene structure and/or single amino acid change in functionally active residues for genes in monoterpenoid, diterpenoid, sesquiterpenoid, and triterpenoid biosynthesis pathways. (**a**) (*3S*)-linalool synthase (TPS14, K15086), (**b**) cytochrome P450, family 82, subfamily G, polypeptide 1 (K17961), (**c**) (+)-neomenthol dehydrogenase (K15095), (**d**) gibberellin 2-oxidase (K04125), and (**e**) squalene monooxygenase (SQLE, ERG1, K00511)

The functional residues of (3*S*)-linalool synthase (MRNA_017575_1) in *M. rubra* contained some variations (Fig. 9a), and those of CYP82G1 (MRNA_015141_1) showed low similarity to orthologs in the other five species (Fig. 9b). However, the expression level of (3*S*)-linalool synthase (K15086) was very low throughout the process of fruit development (Fig. 6c, Additional file 1: Table S14), while CYP82G1 (K17961, MRNA_015141_1) was expressed at an intermediate level. The latter was weakly correlated with the total antioxidant capacity during fruit development (r = 0.408) (Fig. 6d, Additional file 1: Table S15). The phylogenetic tree based on (-)-germacrene D synthase (Fig. 8f) revealed that both the Rosales clade (*M. notabilis*, *P. mume*, and *F. × ananassa*) and the Fagales clade (*M. rubra*) expanded during the course of evolution. This genome expansion may not be entirely clade-specific as the sub-branches of gene trees were expanded in only one species. The (-)-germacrene D synthase (MRNA_026243_1, K15803) gene showed high expression during the first 20 days after pollination and its expression level decreased subsequently. The correlation coefficient between gene expression and total antioxidant capacity was 0.853 (Fig. 6e; Fig. 7e; Additional file 1: Table S16), indicating that the enzyme is important in the early sesquiterpenoid/triterpenoid biosynthesis and in the formation of the total antioxidant capacity in early stage fruit (Fig. 3b).

The genes implicated in monoterpenoid (Fig. 6c), diterpenoid (Fig. 6d), sesquiterpenoid, and triterpenoid (Fig. 6e) biosynthesis pathways were also active in *M. rubra*. For example, K15095 [(+)-neomenthol dehydrogenase, MRNA_022650_1] (Fig. 6c ; Fig. 7f; Additional file 1: Table S14), K04125 (gibberellin 2-oxidase, MRNA_015875_1) (Fig. 6d ; Fig. 7g; Additional file 1: Table S15), and K00511 (squalene monooxygenase, MRNA_011718_2) (Fig. 6e; Fig. 7h; Additional file 1: Table S16) were highly expressed throughout fruit development and respectively participate in monoterpenoid biosynthesis, diterpenoid biosynthesis class metabolism, and sesquiterpenoid and triterpenoid biosynthesis pathways, and they may be important for the total antioxidant capacity during fruit development (Fig. 3b). The functional residues of the (+)-neomenthol dehydrogenase (Fig. 9c), gibberellin 2-oxidase (Fig. 9d), and squalene monooxygenase (Fig. 9e) genes showed low similarity to that in the other five plant species.

Overall, gene analysis indicated that, to obtain Chinese bayberry fruit with high levels of flavonoids, monoterpenoids, diterpenoids, sesquiterpenoids, and triterpenoids and to enhance fruit resistance, CCoAOMT, BZ1, (+)-neomenthol dehydrogenase, gibberellin 2-oxidase, and squalene monooxygenase should be considered in molecular breeding programs.

## Conclusions

This paper reports a compressive genome sequencing study of Chinese bayberry and the transcriptomic analysis of antioxidant pathways in Chinese bayberry fruit. The results obtained provide basis for functional genomic research and genetic improvement of *M. rubra*, and are valuable for inferring evolutionary relationships across related species. The genome sequence provided here, together with the existing genome assembly in the database, are crucial for mining DNA sequence variations between varieties and particular sequences for developing molecular markers to screen Chinese bayberry varieties rich in total flavonoids. Whereas the published genome sequencing project for Chinese bayberry focused on sex determination, this study paid primary attention to the analysis of antioxidant pathways for improved understanding of the resistance mechanisms. Most *M. rubra* genes in the antioxidant signaling pathways have multiple copies via tandem duplication events, and many genes contain structural and amino acid changes of functionally active residues with low similarity to the genes of *F. × ananassa*, *S. lycopersicum*, *M. notabilis*, *P. mume*, and *C. lanatus*. The expression levels of most genes were high during early fruit development and consistent with the high levels of total flavonoids and antioxidant capacity. The genes coding for CCoAOMT, BZ1, (+)-neomenthol dehydrogenase, gibberellin 2-oxidase, and squalene monooxygenase, are suggested as the major regulators of flavonoid, anthocyanin, monoterpenoid, diterpenoid, and sesquiterpenoid/triterpenoid contents, respectively, throughout fruit development. The revealed genome sequences of the representative antioxidant signaling pathways hold great potential for the discovery of useful traits and functional genes in Chinese bayberry.

## Methods

### Plant materials for genome and transcriptome sequencing

Fresh leaves of 10-year-old *M. rubra* ‘Zaojia’ individuals were collected from Lanxi County, Zhejiang Province, China, for genome sequencing. The fruit were not visible to the naked eye until the 10th day after pollination, and therefore all samples were taken on the 10th day and set as reference samples. For transcriptome sequencing, fruit samples were collected 10, 20, 30, 40, and 50 days after the initial pollination event on April 3, 2016. Each sample comprised three biological replicates, which were collected from different trees and consisted of fruit from eastern, western, southern, and northern sides of the tree. Samples were denucleated, ground, immediately frozen in liquid nitrogen, and stored in an ultra-low temperature freezer (Haier 86L-386; Haier, Qingdao, China). These samples were also used for measurements of flavonoid content and total antioxidant capacity.

### DNA extraction and Illumina short paired-end (PE) reads sequencing

Genomic DNA was extracted using the Cetyl trimethylammonium bromide DNA extraction protocol, purified, and quantified using Qubit 2.0 (Invitrogen, Carlsbad, CA, USA). Eleven standard libraries (with insert sizes of 200 bp, 220 bp, 500 bp, 3 kb, 4 kb, 8 kb, 10 kb, 15 kb, and 17 kb) were constructed using Illumina’s paired-end and mate-pair kits (Illumina, San Diego, CA, USA). These libraries were PE sequenced (2 × 150 bp) on the Illumina HiSeq 2500 platform, yielding 64.18 Gb of raw data with 210.75-fold coverage of the *M. rubra* genome.

### Genome assembly and evaluation

A *de novo* assembly pipeline was developed for genome assembly. A whole-genome shotgun assembler ALLPATHS-LG (allpathslg-52488) [40] was used to generate contigs. These contigs were used for scaffolding in SSPACE [41] with reads of long mate-pair libraries. GapCloser 1.12 [42] was used for gap filling. The 14,097 transcripts > 1 kb were assembled using Trinity [43] and aligned to the assembly with BLAT [44]. These procedures, and CEGMA 2.3 [45], were used for genome assembly evaluation.

### Comparative genomics

OrthoMCL 2.0 [46] was used to cluster gene families. All-against-all comparisons of proteins were performed using the basic local alignment search tool (BLAST) with a p-cutoff value of 1e-5. Results were filtered using thresholds with an E-value < 1e-5 and percent match length ≥ 50 % (i.e., 50 % of all possible pairs within the group that were matched through BLAST), and potential in-paralog, ortholog, and co-ortholog pairs were found with the OrthoMCL 2.0 Pairs program. These pairs were grouped based on their relationships using the Markov Cluster Algorithm (MCL, https://micans.org/mcl/) program. The single-copy gene families were identified based on the OrthoMCL clustering results. MUSCLE 3.8 [47] was used to perform the multiple alignments of each single copy gene group. Phylogenetic trees were calculated using the maximum likelihood algorithm of PhyML 4.0 [48]. Divergence time was estimated using CODEML and MCMCTREE, as implemented in the PAML package [49], and substitution rates between gene pairs were calculated with CODEML. The substitution rate of each branch was set in CODEML based on protein sequences; because the global clock model was chosen, substitution rates were the same for all branches. Ambiguous characters and alignment gaps were removed, and the calibration time was obtained from the TimeTree database (http://www.timetree.org). The evolution of gene family size in *M. rubra* and related species was evaluated using CAFE [50] based on the maximum likelihood phylogenetic trees with estimated branch lengths. The ancestral gene family sizes were inferred by CAFE using the probabilistic graphic model (p-value). Smaller p-values in gene families indicated more dramatic changes. The Viterbi algorithm was adopted to identify expanded and contracted gene families.

### Flavonoid content and total antioxidant capacity of *M. rubra* fruits

Each Chinese bayberry fruit sample was dried to a constant weight and ground into fine powder. Flavonoids were extracted from fruit samples using the Plant Flavonoid Kit (LHT-2-G; Suzhou Keming Biological Technology Co. Ltd, Suzhou, China) according to the manufacturer’s instructions. The standard curve for total flavonoid content was: y = 5.02x + 0.0007, R^2^ = 0.9996. The flavonoid content (mg/g dry weight) = (∆A – 0.0007) ÷5.02 ×*V sample* ÷ (*V sample* ÷ *V sample total* × *W*), where ∆A: absorbance at 510 nm of sample against sterilized distilled water, *V* sample total is the volume of extraction liquid (2 mL), *V sample* is the sample volume in the reaction (0.54 mL), and *W* is sample quantity (g).

The total antioxidant capacity of each fruit sample was determined using the Total Antioxidant Capacity Kit (TAOC-2-G; Suzhou Keming Biological Technology Co. Ltd) according to the manufacturer’s instructions. The standard curve for the total antioxidant capacity was: y = 0.638x + 0.0645, R^2^ = 0.9989. Total antioxidant capacity (U/g fresh weight) was obtained as =$\frac{1}{0.6308}$(∆A – 0.0645) ×*V total anti* ÷ (*V sample* ÷ *V sample total* × *W*), where Δ*A is absorbance at 593 nm of sample against sterilized distilled water, V* sample total is the volume of extraction liquid (1 mL), *V total* anti is the total volume of the reaction (1.02 mL), *V sample* is the sample volume in the reaction (0.03 mL), and *W* is sample quantity (g).

### RNA extraction, transcriptome sequencing, assembly, and annotation

The total RNA of *M. rubra* fruit was separately isolated from 15 samples using Trizol reagent (Invitrogen) according to the instructions of the manufacturer. The integrity and concentration of RNA samples were checked in the Agilent 2100 Bioanalyzer (Agilent Technologies, Inc., Santa Clara, CA, USA). Fifteen RNA-sequencing RNA-seq libraries were constructed and then sequenced with in the Illumina HiSeq 2500 platform (Illumina). Raw reads were processed to trim low-quality bases at the ends of reads and adaptors via an in-house script. Low quality reads with Ns accounting for 10 % or more, reads where at least half of the bases scored < 10 in PHRED, and reads with adaptor contamination were discarded. The clean RNA-seq data were then mapped to the genome assembly using Tophat2 2.0.14 [51] to perform reference-guided mapping; the TopHat alignment was assembled using Cufflinks 2.2.1 [52]. The sequencing and mapping statistics are summarized in Table S17.

### Expression profiles of functional genes

Cuffquan and CuffnormGene modules of Cufflinks 2.2.1 were used to quantify the expression levels of individual genes, and fragments per kilobase of transcript per million fragments mapped (FPKM) values served as the measurement. The values were log_2_FPKM transformed, and genes with FPKM < 1 in at least five samples were removed. Expression profiles of functional genes involved in flavonoid, anthocyanin, monoterpenoid, diterpenoid, and sesquiterpenoid/triterpenoid biosynthesis pathways in *M. rubra* are displayed in tables and heat maps. The correlations between functional gene expressions and total flavonoid content or total antioxidant capacity were analyzed using SPSS Statistics 17.0 software. The correlation coefficients were between -1 and +1.

### Homologs of genes and protein sequences

Homologs of genes involved in flavonoid and terpenoid metabolic pathways were detected in *M. rubra*, *M. notabilis*, *P. mume*, *F. × ananassa, C. lanatus*, and *S. lycopersicum*, and multiple sequence alignments of homologs were performed using ClustalW2 (https://www.ebi.ac.uk/Tools/msa/clustalw2/). Phylogenetic analysis of of genes involved in flavonoid and terpenoid metabolic pathways were performed using the neighbor-joining method of MEGA software (Molecular Evolutinary Genetics Analysis, version 6.0). Protein sequences of the genes putatively involved in *M. rubra* pathways were submitted to SWISS-MODEL (https://swissmodel.expasy.org/), and protein structure homology modeling was performed. The protein database consisting of experimentally checked protein structures was searched to select templates (Additional file 1: Table S18). Suitable templates were sorted by predicted global quality estimation scores.

### Quantitative RT-PCR

Complementary DNA sequences were obtained from the total RNA samples using the FastKing RT Kit with gDNase (KR180123) according to the manufacturer’s instructions (TIANGEN Biotech Co., Ltd., Beijing, China). A quantitative PCR was performed with SuperReal PreMix Plus (including SYBR Green) according to the manufacturer’s instructions (FP171206; TIANGEN Biotech Co., Ltd.) on a Roche LightCycler 96 (Roche Molecular Systems, Inc., Pleasanton, CA, USA). The 20-μL total volume reaction comprised 2 × SuperReal PreMix Plus (10 μL), primer solutions (0.6 μL for each primer), cDNA (2 μL), and RNase-free double-distilled water (6.8 μL). The PCR profile included pre-degeneration (95 °C for 30 s, 1 cycle), three-step amplification (95 °C for 5 s, 60 °C for 30 s, and 72 °C for 30 s, 40 cycles), melting (95 °C for 1 s, 65 °C for 15 s, 95 °C for 1 s), and cooling (37 °C for 30 s). The internal reference gene was MRNA_021433_1 (MrUBQ1), and the eight target genes chosen to validate the transcriptome data were MRNA-008272-1 (anthocyanidin 3-O-glucosyltransferase, BZ1, K12930), MRNA-011576-1 (caffeoyl-CoA O-methyltransferase, K005888), MRNA_004300_1 (chalcone synthase, K00660), MRNA_014338_1 (flavonoid 3',5'-hydroxylase, K13083), MRNA_026243_1 ((-)-germacrene D synthase, K15803), MRNA-022650-1 ((+)-neomenthol dehydrogenase, K15095), MRNA_015875_1 (gibberellin 2-oxidase, K04125), and MRNA_011718_2 (squalene monooxygenase, K00511). The relative expression. of the target genes was evaluated using the 2^-ΔΔCt^ method [53], where ΔΔCt = ΔCtT - ΔCtR, and ΔCtT = Ct (test sample, target gene) - Ct (test sample, reference gene), ΔCtR = Ct (reference sample, target gene) - Ct (reference sample, reference gene). The primers used and the product sizes are displayed in Table 1.

**Table 1:** Primers used and the product sizes of the eight target genes examined in the quantitative PCR.

Gene mRNA No.	Left primer	Right primer	Product size
MRNA_008272_1	AACGGACGGATGGTAGAGGA	TGATCTTCTCGCGCATCCTC	136
MRNA_011576_1	AGAGCCTTTTGCAGAGCGAT	GCTCCTTCATGGCTTCAGGT	84
MRNA_004300_1	GCGGCTGCTGTCATAATTGG	AATGTAAGCCCCACCTCACG	140
MRNA_014338_1	CTTCCACCTGGCCCTAAAGG	GAGGCCACGACCATGTTACA	146
MRNA_026243_1	TGATTGACGCAATCCAACGC	TCCACGTGCTTTTTGCTAGC	112
MRNA_022650_1	AGTGTCGCTTCTTTGGCAGA	TCAATTGGAGCTCCTGCCTG	134
MRNA_015875_1	GGGAGGTTCAAGAGCGTGAA	TGGCCCTCCGAAGTAGATCA	81
MRNA_011718_2	CTCTTGGCAAGGATGGACGT	GTAGCAGCTCCCCCACAATT	81
MRNA_021433_1	AAGGCGAAGATCCAAGACAA	GTGGAGCGTCGACTCTTTCT	116

## Additional files

Additional file 1: **Table S1.** *Myrica rubra* genome sequencing data. **Table S2.** *Myrica rubra* genome assembly. **Table S3.** Comparison of the Chinese bayberry genome assembly with other plant genomes. **Table S4.** General statistics of the predicted protein-coding genes. **Table S5.** Statistics of the gene pairs used for fourfold synonymous third-codon transversions (4DTV) distribution analysis. **Table S6.** Statistics of orthologous gene numbers in *Myrica rubra*, *Fragaria ananassa*, *Morus notabilis*, *Solanum lycopersicum*, and *Vitis vinifera*. **Table S7.** Statistics of orthologous gene numbers in *Myrica rubra*, *Cicer arietinum*, *Arabidopsis thaliana*, and *Oryza sativa.* **Table S8.** Annotation of expanded gene families in *Myrica rubra* and in *Solanum lycopersicum*, *Prunus mume*, *Morus notabilis*, *Citrullus lanatus*, and *Fragaria* × *ananassa*. **Table S9.** Annotation of contracted gene families in *Myrica rubra* and in *Solanum lycopersicum*, *Prunus mume*, *Morus notabilis*, *Citrullus lanatus*, and *Fragaria* × *ananassa*. **Table S10**. Total flavonoid content and total antioxidant capacity of Chinese bayberry fruit at specific time points. **Table S11.** Statistics of the gene copies involved in flavonoid biosynthesis, anthocyanin biosynthesis, monoterpenoid biosynthesis, diterpenoid biosynthesis class metabolism, and sesquiterpenoid/triterpenoid biosynthesis pathways. **Table S12.** Expression profiles of functional genes involved in the flavonoid biosynthesis pathways of *Myrica rubra* and correlations with total flavonoid contents and total antioxidant capacities*.* **Table S13.** Expression profiles of functional genes involved in the anthocyanin biosynthesis pathways of *Myrica rubra* and correlations with total flavonoid contents and total antioxidant capacities. **Table S14.** Expression profiles of functional genes involved in the monoterpenoid biosynthesis pathways in *Myrica rubra* and correlations with the total antioxidant capacity. **Table S15.** Expression profiles of functional genes involved in the diterpenoid biosynthesis pathways in *Myrica rubra* and correlations with the total antioxidant capacity. **Table S16.** Expression profiles of functional genes involved in sesquiterpenoid/triterpenoid biosynthesis pathway in *Myrica rubra* and correlations with total antioxidant capacity*.* **Table S17.** Statistics of transcriptome sequencing and mapping. **Table S18.** Summary of protein structure homology modeling in *Myrica rubra*. **Table S19.** Assessment of unigene assembly with *de novo* assembled transcripts. **Table S20.** Assessment of genome assembly by single base error rate. **Table S21.** Evaluation of genome assembly completeness by the Core Eukaryotic Genes Mapping Approach. Table S22. Summary of sequence repeats in the *Myrica rubra* genome. Table S23. Statistics of the functional annotation for genome assembly. Table S24. Statistics of non-coding RNAs and pseudogene annotation. Table S25. KEGG database annotation of *Myrica rubra*. Table S26. Motif and domain statistics detected for *Myrica rubra* in several databases. Table S27. Genes under positive selection in *Myrica rubra*. Table S28. Gene ontology annotation of positively selected genes in *Myrica rubra*. (XLSX 513 kb)

Additional file 2: **Figure S1.** Genome size evaluation and GC content distribution in *Myrica rubra*. **Figure S2.** Gene structure in *Myrica rubra*, *Arabidopsis thaliana*, *Oryza sativa*, and *Vitis vinifera*. **Figure S3.** Synonymous nucleotide substitution (Ks) distribution of homologous gene pairs in *Myrica rubra, Morus notabilis*, and *Vitis vinifera* comparisons. **Figure S4** Genome analysis of *Myrica rubra*, *Cicer arietinum*, *Arabidopsis thaliana*, and *Oryza sativa.* **Figure S5.** *Myrica rubra* phylogenies based on single-copy genes common to other plants. **Figure S6.** Total flavones and colors of the six fruits compared in the present study. **Figure S7.** Divergence distribution of sequence repeats in the *Myrica rubra* and *Morus notabilis* genomes. (DOCX 1660 kb)

## Data Availability

The datasets generated and analyzed during the current study are available in the NCBI database under BioProject PRJNA362655 (https://www.ncbi.nlm.nih.gov/).
