# Supplementary material for: Genome sequencing provides insights into the evolution and antioxidant activity of Chinese bayberry
Source: BMC Genomics. 2019 Jun 6;20:458. doi: 10.1186/s12864-019-5818-7 (PMC6554995; doi:10.1186/s12864-019-5818-7)
Supplement: Supplementary file 2 — Figure S1. Genome size evaluation and GC content distribution in Myrica rubra. Figure S2. Gene structure in Myrica rubra, Arabidopsis thaliana, Oryza sativa, and Vitis vinifera. Figure S3. Synonymous nucleotide substitution (Ks) distribution of homologous gene pairs in Myrica rubra, Morus notabilis, and Vitis vinifera comparisons. Figure S4 Genome analysis of Myrica rubra, Cicer arietinum, Arabidopsis thaliana, and Oryza sativa. Figure S5. Myrica rubra phylogenies based on single-copy genes common to other plants. Figure S6. Total flavones and colors of the six fruits compared in the present study. Figure S7. Divergence distribution of sequence repeats in the Myrica rubra and Morus notabilis genomes. (DOCX 1660 kb) [file 12864_2019_5818_MOESM2_ESM.docx]

**Additional file 2**

1. **Materials and Methods**
   1. **Estimation of genome size with 17-kmer analysis and raw data filtering**

The K-value was set to 17. Low-quality reads (reads with adaptor contamination, average PHRED score < 20%, or > 5% undetermined bases) were discarded before the 17-kmer-frequency analysis. Using high-quality sequencing data, the 17-kmer analysis indicated that the peak frequency of Kmer depth was approximately 37.56. Therefore, the genome size of *Myrica rubra* was estimated as 304.38 Mb. Clean reads were used for assembly.

- 1. **Prediction of repetitive elements**

Repeat sequences in the M. rubra genome were surveyed based on a combination of de novo and homolog searches using RepBase 14.07 [1]. The de novo prediction software MITE-Hunter [2], LTR_FINDER [3], and RepeatScout 1.0.3b [4], and the software for repetitive DNA PILER-DF [5], were used to construct a de novo repeat library. The de novo repeat database was classified using PASTEClassifier (https://urgi.versailles.inra.fr/Tools/PASTEClassifier). This library, combined with the nucleotide repetitive database of RepBase 14.07, served as the searching database. RepeatMasker 4.0.5 [6] was employed to identify and classify the repeated elements. The Kimura two-parameter model and the DistMat software [7] implemented in the EMBOSS package (http://emboss.sourceforge.net/) were used to calculate the distances between long terminal repeats (LTRs), and the LTR insertion event was then dated.

- 1. **Gene model prediction and functional annotation**

Gene prediction was performed using three different strategies to build optimal gene models: (1) The *de novo* gene prediction of the repeat-masked genome was performed using Genscan [8], Augustus 2.4 [9], GlimmerHMM 3.0.3 [10], GeneID 1.2 [11], and SNAP [12]. Genscan was used with the proteins of *Arabidopsis* *thaliana* as the reference using the hidden Markov model (HMM); (2) RNA sequencing (RNA-seq) analysis was performed to enhance the accuracy of gene prediction. The clean reads resulting from RNA-Seq were aligned to the whole genome using Bowtie, as implemented in TopHat 2.0.14 [13], and the mapping results were analyzed to identify splice junctions between exons. Mismatches were not allowed when more than two bases could not be mapped onto the genome. Cufflinks 2.2.1 [14] was used to assemble the alignments into transcripts; (3) Homology-based prediction was performed for the alignment of *A. thaliana* protein sequences to the genome sequence using genBlastA 1.0.1 [15] with an E-value cutoff of *1e-5* and a protein identity of 50 %. The reference proteins of *A. thaliana* were used in GENEWISE 2.2.0 [16] to predict and to filter accurate genes with exon–intron boundaries. Gene model evidence from the above three methods was integrated in GLEAN [17] to produce a high-confidence gene set. The final gene set had gene, coding sequence, exon, and intron length distributions similar to those of other plant species. Gene functions were assigned according to the best match of the alignments against various protein databases using the basic local alignment search tool (BLAST; E-value = 1e-5) on the National Center for Biotechnology Information non-redundant (NR) protein [18], non-redundant nucleotide sequence, Swiss-Prot [19], InterProScan [20], Kyoto Encyclopedia of Genes and Genomes (KEGG) [21], Clusters of Orthologous Groups (COG) [22], KOG (ftp://ftp.ncbi.nih.gov/pub/COG/KOG/kog), TrEMBL(http://www.bioinfo.pte.hu/more/TrEMBL.htm) and GO (http://geneontology.org/) databases to predict and classify gene functions.

- 1. **Prediction of non-coding RNAs**

The prediction of ribosomal and micro RNA (rRNA and miRNA, respectively) sequences was based on BLAST against the Rfam database [23]. The software tRNAscan-SE 1.23 [24] with eukaryote parameters was used to identify transfer RNA (tRNA) positions.

- 1. **Whole genome duplication analysis**

MCscan 0.8 [25] was used to search syntenic blocks with a distance cutoff value of 20 genes, and gene pairs were aligned based on at least five gene pairs per syntenic block. In the alignment of genes against their own genome, each syntenic block indicates the paralogous gene pairs that result from genome duplication. In inter-species alignments, each syntenic block represents the orthologous gene pairs from recently shared ancestors. Speciation and genome duplications in the evolutionary history of *M. rubra* were assessed by calculating the distribution of fourfold synonymous third-codon transversions for syntenic blocks.

- 1. **Positively selected genes**

The positively selected genes in *M. rubra* were identified using the branch-site model and the likelihood ratio test implemented in the CODEML program of PAML 4.8a [26]. *Myrica* *rubra* was placed on the foreground branch and the other species on the background branches.

- 1. **Gene family analysis**

Protein sequences of *A. thaliana* were used as references to search for homologous protein candidates, considering 50 % coverage and 50 % identity. Conserved domains were detected using the Pfam database [27], and genes without conserved domains shared by the gene family were removed. The candidate genes were annotated in KEGG [21]. Sequence similarity and conserved domains were considered.

- 1. **Identification of genes encoding nucleotide-binding site (NBS) proteins**

Most NBS proteins are characterized by C-terminal leucine-rich repeat (LRR) sequences (NBS-LRR) and either Toll/interleukin-1 receptor (TIR) sequences or coiled-coil (CC) motifs at the N-terminus of a protein. Using HMMER 3.0 [28], The Pfam NBS domain PF00931 (NB-ARC domain) was detected. The predicted NBS proteins were further screened for the presence of TIR (PF01582) and LRR motifs (PF00560, PF07723, and PF07725) using SMART [29] and Pfam [30] databases, respectively. COILS [31] was used to detect CC domains with a threshold probability of 90 %.

1. **Results and discussion**
   1. **Genome assembly and annotation**

The GC content was 37.5 % across the genome and 46 % in the coding sequences (Additional file 1: Table S2; Additional file 2: Figure S1b). Most (97.11 % ) of the unigenes longer than 1 kb (14,097 in total) were mapped to the assembled genome (Additional file 1: Table S19). The comparison of the sequenced reads and assembled genome sequence indicated a low error rate (0.0005 %) for the total contig length (Additional file 1: Table S20). Among the 248 conserved core eukaryotic genes that were used to assess genome completeness, 246 (99.19 %) were found in our genome assembly (Additional file 1: Table S21), and most transcripts were mapped to the genome assembly.

In total, 132.24 Mb of repetitive sequences (45.61 % of the assembly) were identified in the Chinese bayberry genome (Additional file 1: Table S22). Most of these repetitive sequences (89.34 %) could be classified and annotated; the remaining 10.66 %, which may be Chinese bayberry-specific, remained unclassified (Additional file 1: Table S22). The LTR family was the most abundant (total length 58,887,942 bp, accounting for 20.31 % of the genome) (Additional file 1: Table S22). Within the LTR family, Copia and Gypsy represented the two most abundant subfamilies (Additional file 1: Table S22). In addition, DNA transposons accounted for 13.71 % of the genome assembly (39.75 Mb) (Additional file 1: Table S22). The most active time for LTR inserts was estimated at 2.5–3.7 Mya, and abundant LTR insertions occurred recently in a narrow time window in *M. rubra* and *Morus notabilis* (Additional file 2: Figure S7).

Additionally, 56.81, 94.38, 19.78, 94.49, 34.07, 55.13, 74.25, and 68.48 % of the genes were annotated using the KOG, TrEMBL, KEGG, NR, COG, GO, Pfam, and Swiss-Prot databases, respectively (Additional file 1: Table S23), as well as 577 miRNAs, 149 rRNAs, 248 tRNAs, and 2,726 predicted pseudogenes (Additional file 1: Table S24).

- 1. **Comparative genome analysis**

One hundred and seventeen metabolic pathways were identified in Chinese bayberry (Additional file 1: Table S25). The genes participating in six pathways related to antioxidant effects were identified, namely those involved in terpenoid backbone biosynthesis (81), ubiquinone and other terpenoid-quinone biosynthesis (69), steroid biosynthesis (57), flavonoid biosynthesis (34), diterpenoid biosynthesis (12), and flavone and flavonol biosynthesis (12) (Additional file 1: Table S25). Among the 1,075 motifs and 3,555 domains identified, the RNA recognition motif (356) and serine/threonine protein kinase catalytic domains (646) were the most common (Additional file 1: Table S26).

- 1. **Positive selection**

The ratio of non-synonymous to synonymous substitutions per gene (ɷ) was estimated from the alignments of 1,738 single-copy genes using the CODEML program under a branch-site model and F3 × 4 codon frequencies (Additional file 1: Table S27). A likelihood ratio test was subsequently performed, and 114 positively selected genes were identified by means of false discovery rate adjustment with *p* < 0.05 (Additional file 1: Table S28). The most positive selection pressures was related to replication, recombination and repair, intracellular trafficking, secretion, vesicular transport, coenzyme transport and metabolism, translation, and ribosomal structure and biogenesis, indicating that they might have been involved in Chinese bayberry adaptations to the environment (Additional file 1: Table S28).

- 1. **Gene structure and/or single amino acid changes in functionally active residues**

The protein structures of the genes involved in the flavonoid metabolic pathway were modeled. The most suitable template for *MRNA_017263_1* (anthocyanidin 3-O-glucoside 5-O-glucosyltransferase, UGT75C1) was *A. thaliana* uridine diphosphate–glycosyltransferase 74F2 (UGT74F2) [32]. In *A. thaliana*, the uridine diphosphate–glucose binding sites of UGT74F2 were residues 332–362 [32]. Despite being 77 % identical and with conserved active site residues, UGT74F1 and UGT74F2 catalyze the formation of different products: UGT74F1 forms salicylic acid glucoside, whereas UGT74F2 primarily forms salicylic acid glucose esters. The position of the glucose on the aglycone determines how salicylic acid is stored, metabolized, and contributes to a defense response [32]. In *M. rubra* (UGT75C1), residues 339–369 were the active binding sites (Figure 4b). Although most of these residues were highly similar in the five species, some (S345, H346, and T361) differed (Figure 4b). The most suitable template for *MRNA-011576-1* (Caffeoyl coenzyme A 3-O-methyltransferase; CCoAOMT) was *Medicago sativa* CCoAOMT [33]. The overall homology similarity among CCoAOMTs from different species was high. In particular, the residues involved in substrate recognition on eight helices were highly conserved among the CCoAOMTs [33] derived from different species (Figure 4d). The best template for *MRNA_004300_1* (chalcone synthase, CHS) was CHS from *Malus* × *domestica* (MdCHS2) [34]. The overall similarity among CHSs derived from different sources was also high (Figure 4e). The gatekeeper residues Phe215 and Phe265 in MdCHS2 affected substrate selectivity. The active-site cavity composed of Ser133, Glu192, Thr194, Thr197, Gly216, and Ser338 is involved in substrate loading [34]. The top template for *MRNA_017633_1* was the anthocyanidin reductase from grapevine (*Vitis vinifera*) [35]. The β1α1 motif, a glycine-rich loop comprising Gly16-Gly17-Thr18-Gly19-Phe20-Val21-Ala22-Ser23 (residues at the NADPH-binding site), is predicted to bind to the cofactor. In *M. rubra* and *M. notabilis*, Thr18 was substituted by Ser18 (Figure 4f). The optimal template for *MRNA_020916_2* (chalcone isomerase, CHI) was AtCHI (At3g55120) [36]. Catalytic residues in *M. rubra* were Arg38, Thr50, Tyr108, Asn115, and Thr190. In *M. rubra*, *M. notabilis*, *P. mume*, *F. × ananassa*, and *Citrullus lanatus*, these residues were conserved (Figure 4g). In *S. lycopersicum*, Asn115 and Thr190 were not conserved and were respectively substituted by Ser50 and Phe108 (Figure 4g). The best template for *MRNA_001369_2* and *MRNA_001461_1* (bifunctional dihydroflavonol 4-reductase/flavanone 4-reductase, DFR) was *V. vinifera* DFR [37]. This is a key enzyme in the flavonoid biosynthesis pathway and mediates the reduction of 2R, 3R-trans-dihydroflavonols to leuco-anthocyanidins. In *V. vinifera* DFR, the catalytic residues involved in ligand–protein interactions are Ser128, Asn133, Tyr163, and Gln227 [37]. The residue Asn133 was not conserved and was substituted by Asp133 in the *MRNA_001369_2* of *S. lycopersicum* and *M. notabilis* (Figure 4h). In *P. mume*, the residue was deleted (Figure 4h). The top template for *MRNA_009502_1* (leucoanthocyanidin dioxygenase) was *A. thaliana* anthocyanidin synthase [38]. Residues involved in enzymatic activity were conserved in the analyzed species (Figure 4i). The optimal template for *MRNA_012654_1* (leucoanthocyanidin reductase, LAR) was *V. vinifera* LAR [39]. This enzyme is involved in the flavonoid metabolic pathway by catalyzing the NADPH-dependent reduction of 2R, 3S, 4S-flavan-3,4-diols into 2R, 3S-flavan-3-ols, which are important for plant survival. In *V. vinifera* LAR, the side chains of His122, Tyr137, Lys140, and Ser161 could also be involved in the catalytic mechanism. These residues were conserved in *M. rubra*, *M. notabilis*, *F. ananassa*, and *P. mume* (Figure 4j). The templates for shikimate O-hydroxycinnamoyltransferase (HCT, K13065) [40] (Figure 4a), anthocyanidin 3-O-glucosyltransferase (*MRNA_008272_1*) [41] (Figure 4c), flavonol synthase (*MRNA_021063_1*) [42] (Figure 4k), naringenin 3-dioxygenase (*MRNA_013165_1*) [44] (Figure 4l), CYP73A (*MRNA_010278_2*) [43] (Figure 4m), flavonoid 3',5'-hydroxylase (*MRNA_014338_1*) [44] (Figure 4n), and flavonoid 3'-monooxygenase (*MRNA_009278_3*) [45] (Figure 4o) were identified in the database, but their low similarity among species hampered further analysis.

The protein structures of the genes involved in the terpenoid metabolic pathway were also modeled. The most suitable templates for *MRNA_017575_1* ((*3S*)-linalool synthase, TPS14) were the *Populus* × *canescens* terpenoid cyclases [42]. For terpenoid cyclases, the metal-binding motifs (the “aspartate-rich” motif D345DXXD and the “NSE/DTE” motif N489DXXSXXXE) in the catalytic domain (positions for *P.* × *canescens*) were conserved in plants [42]. The aspartate motifs in the five species were the same as the conserved residues (Figure 8a). For the NSE/DTE motif, the first residue was D instead of the canonical N (Figure 8a) in the five species. In *M. rubra*, the D476, S479, and E483 residues of the NSE/DTE motif were of functional significance. Although sequence alignment and homology modeling showed that the NSE/STE motifs of *M. rubra*, *M. notabilis*, *P. mume*, and *F. × ananassa* were highly conserved, the T479 residue in *C. lanatus* differed from the S479 of other species (Figure 8a). This motif is essential for the departure of the dimethylallyl diphosphate group in isoprene biosynthesis. Cytochrome P450, family 82, subfamily G, polypeptide 1 (*MRNA_015141_1*) [43] (Figure 8b), (+)-neomenthol dehydrogenase (*MRNA_022650_1*) [46] (Figure 8c), gibberellin 2-oxidase (*MRNA_015875_1*) [47] (Figure 8d), and SQLE, ERG1 (*MRNA_011718_2*) [48] (Figure 8e) were not analyzed further due to their low similarity to the templates.

- 1. **Flavonoid content and total antioxidant capacity of Chinese bayberry during fruit development**

During the growth and development of Chinese ‘Zaoiia’ bayberry fruit, the flavonoid content (Figure 6a) and the total antioxidant capacity (Figure 6b) gradually decreased, with the former decreasing faster than the latter. The flavonoid content in fruit remained at high levels, namely 50.26–52.51 mg/g dry weight between 10 and 20 days after pollination. These patterns mirrored the expression patterns of the genes related to flavonoid biosynthesis pathways (Figure 5a and 5b). The antioxidant capacity of fruit decreased slowly over the 50 days after pollination, from 1241.59 to 995.62 U/g fresh weight, and it was coincident with the expression patterns of genes related to monoterpenoid (Figure 5c), diterpenoid (Figure 5d), and sesquiterpenoid/triterpenoid (Figure 5e) biosynthesis pathways.

1. **Supplemental References**
2. Gnerre S, [Maccallum I](https://www.ncbi.nlm.nih.gov/pubmed/?term=Maccallum%20I%5BAuthor%5D&cauthor=true&cauthor_uid=21187386), [Przybylski D](https://www.ncbi.nlm.nih.gov/pubmed/?term=Przybylski%20D%5BAuthor%5D&cauthor=true&cauthor_uid=21187386), [Ribeiro F](https://www.ncbi.nlm.nih.gov/pubmed/?term=Ribeiro%20FJ%5BAuthor%5D&cauthor=true&cauthor_uid=21187386), [Burton J](https://www.ncbi.nlm.nih.gov/pubmed/?term=Burton%20JN%5BAuthor%5D&cauthor=true&cauthor_uid=21187386), [Walker B](https://www.ncbi.nlm.nih.gov/pubmed/?term=Walker%20BJ%5BAuthor%5D&cauthor=true&cauthor_uid=21187386), et al. High-quality draft assemblies of mammalian genomes from massively parallel sequence data. Pro Natl Acad Sci USA*.* 2011;108:1513-8.
3. Boetzer M, Henkel C, Jansen H, Butler D, Pirovano W. Scaffolding pre-assembled contigs using SSPACE. Bioinformatics. 2011;27:578-9.
4. Luo R, Liu B, Xie Y, Li Z, Huang W, Yuan J, et al. SOAPdenovo2: an empirically improved memory-efficient short-read de novo assembler. Gigascience. 2012;1:18.
5. Grabherr M, Haas B, Yassour M, Levin J, Thompson D, Amit I, et al. Full-length transcriptome assembly from RNA-seq data without a reference genome. Nat Biotechnol. 2011;29:644-52.
6. Kent W. BLAT -The BLAST-Like Alignment Tool. Genome Res. 2002;12:656-64.
7. Parra G, Bradnam K, Korf I. CEGMA: a pipeline to accurately annotate core genes in eukaryotic genomes. Bioinformatics. 2007;23:1061-7.
8. Jurka J, Kapitonov V, Pavlicek A, Klonowski P, Kohany O, Walichiewicz J. Repbase Update, a database of eukaryotic repetitive elements. Cytogenet Genome Res*.* 2005;110:462-7.
9. Han Y, Wessler S. MITE-Hunter: a program for discovering miniature inverted-repeat transposable elements from genomic sequences. Nucleic Acids Res. 2010;38:e199.
10. Xu Z, Wang H. LTR_FINDER: an efficient tool for the prediction of full-length LTR retrotransposons. Nucleic Acids Res*.* 2007;35:W265-8.
11. Price A, Jones N, Pevzner P. De novo identification of repeat families in large genomes. Bioinformatics. 2005;21:351-8.
12. Edgar R, Myers E. PILER: identification and classification of genomic repeats. Bioinformatics. 2005;21:351-8.
13. Tarailo-Graovac M, Chen N. Using RepeatMasker to identify repetitive elements in genomic sequences. [Curr Protoc Bioinformatics.](https://www.ncbi.nlm.nih.gov/pubmed/19274634) 2009;Chapter 4:Unit 4.10.
14. Rice P, Longden I, Bleasby A. EMBOSS: The European molecular biology open software suite. *Trends Genet.* 2000;**16**, 276-277.
15. Burge C, Karlin S. Prediction of complete gene structures in human genomic DNA*.* J Mol Biol. 1997;268:78-94.
16. Stanke M, Waack S. Gene prediction with a hidden Markov model and a new intron submodel. Bioinformatics. 2003;19:215-25.
17. Majoros W, Pertea M, Salzberg S. TigrScan and GlimmerHMM: two open source ab initio eukaryotic gene-finders. Bioinformatics. 2004;20:2878-9.
18. Blanco E, Parra G, Guigó R. Using geneid to identify genes. Curr Protoc Bioinformatics. 2007;Chapter 4:Unit 4.3.
19. Korf I. Gene finding in novel genomes. BMC bioinformatics. 2004;5:59.
20. Trapnell C, Roberts A, Goff L, Pertea G, Kim D, Kelley D, et al. Differential gene and transcript expression analysis of RNA-seq experiments with TopHat and Cufflinks. Nat Protoc. 2012;7:562-78.
21. Trapnell C, Pachter L, Salzberg S. TopHat: discovering splice junctions with RNA-Seq. Bioinformatics. 2009;25:1105-11.
22. She R, [Chu J](https://www.ncbi.nlm.nih.gov/pubmed/?term=Chu%20JS%5BAuthor%5D&cauthor=true&cauthor_uid=21653517), [Uyar B](https://www.ncbi.nlm.nih.gov/pubmed/?term=Uyar%20B%5BAuthor%5D&cauthor=true&cauthor_uid=21653517), [Wang J](https://www.ncbi.nlm.nih.gov/pubmed/?term=Wang%20J%5BAuthor%5D&cauthor=true&cauthor_uid=21653517), [Wang K](https://www.ncbi.nlm.nih.gov/pubmed/?term=Wang%20K%5BAuthor%5D&cauthor=true&cauthor_uid=21653517), [Chen N](https://www.ncbi.nlm.nih.gov/pubmed/?term=Chen%20N%5BAuthor%5D&cauthor=true&cauthor_uid=21653517). GenBlastG: using BLAST searches to build homologous gene models. Bioinformatics*.* 2011;27:2141-3.
23. Birney E, Clamp M, Durbin R. GeneWise and genomewise. Genome Res*.* 2004;14:988-95.
24. Elsik C, [Mackey A](https://www.ncbi.nlm.nih.gov/pubmed/?term=Mackey%20AJ%5BAuthor%5D&cauthor=true&cauthor_uid=17241472), [Reese J](https://www.ncbi.nlm.nih.gov/pubmed/?term=Reese%20JT%5BAuthor%5D&cauthor=true&cauthor_uid=17241472), [Milshina N](https://www.ncbi.nlm.nih.gov/pubmed/?term=Milshina%20NV%5BAuthor%5D&cauthor=true&cauthor_uid=17241472), [Roos D](https://www.ncbi.nlm.nih.gov/pubmed/?term=Roos%20DS%5BAuthor%5D&cauthor=true&cauthor_uid=17241472), [Weinstock G](https://www.ncbi.nlm.nih.gov/pubmed/?term=Weinstock%20GM%5BAuthor%5D&cauthor=true&cauthor_uid=17241472). Creating a honey bee consensus gene set. Genome Biol. 2007;8:R13.
25. Marchler-Bauer A, Lu S, Anderson J, Chitsaz F, Derbyshire M, DeWeese-Scott C, et al. CDD: a conserved domain database for the functional annotation of proteins. Nucleic Acids Res. 2011;39:D225-9.
26. Boeckmann B, [Bairoch A](https://www.ncbi.nlm.nih.gov/pubmed/?term=Bairoch%20A%5BAuthor%5D&cauthor=true&cauthor_uid=12520024), [Apweiler R](https://www.ncbi.nlm.nih.gov/pubmed/?term=Apweiler%20R%5BAuthor%5D&cauthor=true&cauthor_uid=12520024), [Blatter M](https://www.ncbi.nlm.nih.gov/pubmed/?term=Blatter%20MC%5BAuthor%5D&cauthor=true&cauthor_uid=12520024), [Estreicher A](https://www.ncbi.nlm.nih.gov/pubmed/?term=Estreicher%20A%5BAuthor%5D&cauthor=true&cauthor_uid=12520024), [Gasteiger E](https://www.ncbi.nlm.nih.gov/pubmed/?term=Gasteiger%20E%5BAuthor%5D&cauthor=true&cauthor_uid=12520024), et al. The SWISS-PROT protein knowledgebase and its supplement TrEMBL in 2003. Nucleic Acids Res. 2003;31:365-70.
27. Zdobnov E, Apweiler R. InterProScan-an integration platform for the signature-recognition methods in InterPro. Bioinformatics. 2001;17:847-8.
28. Kanehisa M, Goto S. KEGG: kyoto encyclopedia of genes and genomes. Nucleic Acids Res. 2000;28:27-30.
29. Tatusov R, Natale D, Garkavtsev I, Tatusova T, Shankavaram U, Rao B, et al. The COG database: new developments in phylogenetic classification of proteins from complete genomes. Nucleic Acids Res. 2001;29:22-8.
30. Griffiths-Jones S, [Moxon S](https://www.ncbi.nlm.nih.gov/pubmed/?term=Moxon%20S%5BAuthor%5D&cauthor=true&cauthor_uid=15608160), [Marshall M](https://www.ncbi.nlm.nih.gov/pubmed/?term=Marshall%20M%5BAuthor%5D&cauthor=true&cauthor_uid=15608160), [Khanna A](https://www.ncbi.nlm.nih.gov/pubmed/?term=Khanna%20A%5BAuthor%5D&cauthor=true&cauthor_uid=15608160), [Eddy S](https://www.ncbi.nlm.nih.gov/pubmed/?term=Eddy%20SR%5BAuthor%5D&cauthor=true&cauthor_uid=15608160), [Bateman A](https://www.ncbi.nlm.nih.gov/pubmed/?term=Bateman%20A%5BAuthor%5D&cauthor=true&cauthor_uid=15608160). Rfam: annotating non-coding RNAs in complete genomes. Nucleic Acids Res*.* 2005;33:D121-4.
31. Lowe T, Eddy S. tRNAscan-SE: a program for improved detection of transfer RNA genes in genomic sequence. Nucleic Acids Res. 1997;25:955-64.
32. Wang Y, [Tang H](https://www.ncbi.nlm.nih.gov/pubmed/?term=Tang%20H%5BAuthor%5D&cauthor=true&cauthor_uid=22217600), [Debarry J](https://www.ncbi.nlm.nih.gov/pubmed/?term=Debarry%20JD%5BAuthor%5D&cauthor=true&cauthor_uid=22217600), [Tan X](https://www.ncbi.nlm.nih.gov/pubmed/?term=Tan%20X%5BAuthor%5D&cauthor=true&cauthor_uid=22217600), [Li J](https://www.ncbi.nlm.nih.gov/pubmed/?term=Li%20J%5BAuthor%5D&cauthor=true&cauthor_uid=22217600), [Wang X](https://www.ncbi.nlm.nih.gov/pubmed/?term=Wang%20X%5BAuthor%5D&cauthor=true&cauthor_uid=22217600), et al. MCScanX: a toolkit for detection and evolutionary analysis of gene synteny and collinearity. Nucleic Acids Res*.* 2012;40:e49.
33. Schabauer H, Valle M, Pacher C, Stockinger H, Stamatakis A, Robinsonrechavi M, [et](https://www.ncbi.nlm.nih.gov/pubmed/?term=Salamin%20N%5BAuthor%5D&cauthor=true&cauthor_uid=24389654) al. SlimCodeML: An optimized version of CodeML for the Branch-Site Model. In IPDPS Workshops. 2012;706-14.
34. Finn R, [Coggill P](https://www.ncbi.nlm.nih.gov/pubmed/?term=Coggill%20P%5BAuthor%5D&cauthor=true&cauthor_uid=26673716), [Eberhardt R](https://www.ncbi.nlm.nih.gov/pubmed/?term=Eberhardt%20RY%5BAuthor%5D&cauthor=true&cauthor_uid=26673716), [Eddy S](https://www.ncbi.nlm.nih.gov/pubmed/?term=Eddy%20SR%5BAuthor%5D&cauthor=true&cauthor_uid=26673716), [Mistry J](https://www.ncbi.nlm.nih.gov/pubmed/?term=Mistry%20J%5BAuthor%5D&cauthor=true&cauthor_uid=26673716), [Mitchell A](https://www.ncbi.nlm.nih.gov/pubmed/?term=Mitchell%20AL%5BAuthor%5D&cauthor=true&cauthor_uid=26673716), et al. The Pfam protein families database: towards a more sustainable future. Nucleic Acids Res. 2016;44:D279-85.
35. Marchin M, Kelly P, Fang J. Tracker: continuous HMMER and BLAST searching. Bioinformatics. 2005;21:388-9.
36. Letunic I, [Copley R](https://www.ncbi.nlm.nih.gov/pubmed/?term=Copley%20RR%5BAuthor%5D&cauthor=true&cauthor_uid=14681379), [Schmidt S](https://www.ncbi.nlm.nih.gov/pubmed/?term=Schmidt%20S%5BAuthor%5D&cauthor=true&cauthor_uid=14681379), [Ciccarelli F](https://www.ncbi.nlm.nih.gov/pubmed/?term=Ciccarelli%20FD%5BAuthor%5D&cauthor=true&cauthor_uid=14681379), [Doerks T](https://www.ncbi.nlm.nih.gov/pubmed/?term=Doerks%20T%5BAuthor%5D&cauthor=true&cauthor_uid=14681379), [Schultz J](https://www.ncbi.nlm.nih.gov/pubmed/?term=Schultz%20J%5BAuthor%5D&cauthor=true&cauthor_uid=14681379), et al. SMART 4.0: towards genomic data integration. Nucleic Acids Res. 2004;32:D142-4.
37. Finn R, [Mistry J](https://www.ncbi.nlm.nih.gov/pubmed/?term=Mistry%20J%5BAuthor%5D&cauthor=true&cauthor_uid=16381856), [Schuster-Böckler B](https://www.ncbi.nlm.nih.gov/pubmed/?term=Schuster-B%C3%B6ckler%20B%5BAuthor%5D&cauthor=true&cauthor_uid=16381856), [Griffiths-Jones S](https://www.ncbi.nlm.nih.gov/pubmed/?term=Griffiths-Jones%20S%5BAuthor%5D&cauthor=true&cauthor_uid=16381856), [Hollich V](https://www.ncbi.nlm.nih.gov/pubmed/?term=Hollich%20V%5BAuthor%5D&cauthor=true&cauthor_uid=16381856), [Lassmann T](https://www.ncbi.nlm.nih.gov/pubmed/?term=Lassmann%20T%5BAuthor%5D&cauthor=true&cauthor_uid=16381856), et al. Pfam: clans, web tools and services. Nucleic Acids Res. 2006;34:D247-51.
38. Lupas A, Van Dyke M, Stock J. Predicting coiled coils from protein sequences. Science*.* 1991;252:1162-4.
39. [George Thompson A](https://www.ncbi.nlm.nih.gov/pubmed/?term=George%20Thompson%20AM%5BAuthor%5D&cauthor=true&cauthor_uid=28425481), [Iancu C](https://www.ncbi.nlm.nih.gov/pubmed/?term=Iancu%20CV%5BAuthor%5D&cauthor=true&cauthor_uid=28425481), [Neet K](https://www.ncbi.nlm.nih.gov/pubmed/?term=Neet%20KE%5BAuthor%5D&cauthor=true&cauthor_uid=28425481), [Dean J](https://www.ncbi.nlm.nih.gov/pubmed/?term=Dean%20JV%5BAuthor%5D&cauthor=true&cauthor_uid=28425481), [Choe J](https://www.ncbi.nlm.nih.gov/pubmed/?term=Choe%20JY%5BAuthor%5D&cauthor=true&cauthor_uid=28425481). Differences in salicylic acid glucose conjugations by UGT74F1 and UGT74F2 from *Arabidopsis thaliana*. [Sci Rep.](https://www.ncbi.nlm.nih.gov/pubmed/?term=Differences+in+salicylic+acid+glucose+conjugations+by+UGT74F1+and+UGT74F2+from+Arabidopsis+thaliana) 2017;7:46629.
40. Ferrer, J, [Zubieta C](https://www.ncbi.nlm.nih.gov/pubmed/?term=Zubieta%20C%5BAuthor%5D&cauthor=true&cauthor_uid=15734921), [Dixon R](https://www.ncbi.nlm.nih.gov/pubmed/?term=Dixon%20RA%5BAuthor%5D&cauthor=true&cauthor_uid=15734921), [Noel J](https://www.ncbi.nlm.nih.gov/pubmed/?term=Noel%20JP%5BAuthor%5D&cauthor=true&cauthor_uid=15734921). Crystal structures of alfalfa caffeoyl coenzyme A 3-O-Methyltransferase. [Plant Physiol.](https://www.ncbi.nlm.nih.gov/pubmed/?term=Crystal+Structures+of+Alfalfa+Caffeoyl+Coenzyme+A+3-O-Methyltransferase) 2005;137(3):1009-17.
41. [Stewart C](https://www.ncbi.nlm.nih.gov/pubmed/?term=Stewart%20C%20Jr%5BAuthor%5D&cauthor=true&cauthor_uid=29199980), [Woods K](https://www.ncbi.nlm.nih.gov/pubmed/?term=Woods%20K%5BAuthor%5D&cauthor=true&cauthor_uid=29199980), [Macias G](https://www.ncbi.nlm.nih.gov/pubmed/?term=Macias%20G%5BAuthor%5D&cauthor=true&cauthor_uid=29199980), [Allan A](https://www.ncbi.nlm.nih.gov/pubmed/?term=Allan%20AC%5BAuthor%5D&cauthor=true&cauthor_uid=29199980), [Hellens R](https://www.ncbi.nlm.nih.gov/pubmed/?term=Hellens%20RP%5BAuthor%5D&cauthor=true&cauthor_uid=29199980), [Noel J](https://www.ncbi.nlm.nih.gov/pubmed/?term=Noel%20JP%5BAuthor%5D&cauthor=true&cauthor_uid=29199980). Molecular architectures of benzoic acid-specific type III polyketide synthases. [Acta Crystallogr D Struct Biol.](https://www.ncbi.nlm.nih.gov/pubmed/?term=Molecular+architectures+of+benzoic+acid-specific+type+III+polyketide+synthases) 2017;73:1007-19.
42. [Gargouri M](https://www.ncbi.nlm.nih.gov/pubmed/?term=Gargouri%20M%5BAuthor%5D&cauthor=true&cauthor_uid=19690377), [Manigand C](https://www.ncbi.nlm.nih.gov/pubmed/?term=Manigand%20C%5BAuthor%5D&cauthor=true&cauthor_uid=19690377), [Maugé C](https://www.ncbi.nlm.nih.gov/pubmed/?term=Maug%C3%A9%20C%5BAuthor%5D&cauthor=true&cauthor_uid=19690377), [Granier T](https://www.ncbi.nlm.nih.gov/pubmed/?term=Granier%20T%5BAuthor%5D&cauthor=true&cauthor_uid=19690377), [Langlois d'Estaintot B](https://www.ncbi.nlm.nih.gov/pubmed/?term=Langlois%20d'Estaintot%20B%5BAuthor%5D&cauthor=true&cauthor_uid=19690377), et al. Structure and epimerase activity of anthocyanidin reductase from *Vitis vinifera*. [Acta Crystallogr D Biol Crystallogr.](https://www.ncbi.nlm.nih.gov/pubmed/?term=Structure+and+epimerase+activity+of+anthocyanidin+reductase+from+Vitis+vinifera) 2009;65:989-1000.
43. [Ngaki M](https://www.ncbi.nlm.nih.gov/pubmed/?term=Ngaki%20MN%5BAuthor%5D&cauthor=true&cauthor_uid=22622584), [Louie G](https://www.ncbi.nlm.nih.gov/pubmed/?term=Louie%20GV%5BAuthor%5D&cauthor=true&cauthor_uid=22622584), [Philippe R](https://www.ncbi.nlm.nih.gov/pubmed/?term=Philippe%20RN%5BAuthor%5D&cauthor=true&cauthor_uid=22622584), [Manning G](https://www.ncbi.nlm.nih.gov/pubmed/?term=Manning%20G%5BAuthor%5D&cauthor=true&cauthor_uid=22622584), [Pojer F](https://www.ncbi.nlm.nih.gov/pubmed/?term=Pojer%20F%5BAuthor%5D&cauthor=true&cauthor_uid=22622584), [Bowman M](https://www.ncbi.nlm.nih.gov/pubmed/?term=Bowman%20ME%5BAuthor%5D&cauthor=true&cauthor_uid=22622584), et al. Evolution of the chalcone-isomerase fold from fatty-acid binding to stereospecific catalysis. [Nature.](https://www.ncbi.nlm.nih.gov/pubmed/?term=Evolution+of+the+chalcone-isomerase+fold+from+fatty-acid+binding+to+stereospecific+catalysis) 2012;485(7399):530-3.
44. [Trabelsi N](https://www.ncbi.nlm.nih.gov/pubmed/?term=Trabelsi%20N%5BAuthor%5D&cauthor=true&cauthor_uid=18645237), [Petit P](https://www.ncbi.nlm.nih.gov/pubmed/?term=Petit%20P%5BAuthor%5D&cauthor=true&cauthor_uid=18645237), [Manigand C](https://www.ncbi.nlm.nih.gov/pubmed/?term=Manigand%20C%5BAuthor%5D&cauthor=true&cauthor_uid=18645237), [Langlois d'Estaintot B](https://www.ncbi.nlm.nih.gov/pubmed/?term=Langlois%20d'Estaintot%20B%5BAuthor%5D&cauthor=true&cauthor_uid=18645237), [Granier T](https://www.ncbi.nlm.nih.gov/pubmed/?term=Granier%20T%5BAuthor%5D&cauthor=true&cauthor_uid=18645237), [Chaudière J](https://www.ncbi.nlm.nih.gov/pubmed/?term=Chaudi%C3%A8re%20J%5BAuthor%5D&cauthor=true&cauthor_uid=18645237), [et](https://www.ncbi.nlm.nih.gov/pubmed/?term=Gallois%20B%5BAuthor%5D&cauthor=true&cauthor_uid=18645237) al. Structural evidence for the inhibition of grape dihydroflavonol 4-reductase by flavonols. [Acta Crystallogr D Biol Crystallogr.](https://www.ncbi.nlm.nih.gov/pubmed/?term=Structural+evidence+for+the+inhibition+of+grape+dihydroflavonol+4-reductase+by+flavonols) 2008;D64:883-91.
45. [Welford R](https://www.ncbi.nlm.nih.gov/pubmed/?term=Welford%20RW%5BAuthor%5D&cauthor=true&cauthor_uid=16106293), [Clifton I](https://www.ncbi.nlm.nih.gov/pubmed/?term=Clifton%20IJ%5BAuthor%5D&cauthor=true&cauthor_uid=16106293), [Turnbull J](https://www.ncbi.nlm.nih.gov/pubmed/?term=Turnbull%20JJ%5BAuthor%5D&cauthor=true&cauthor_uid=16106293), [Wilson S](https://www.ncbi.nlm.nih.gov/pubmed/?term=Wilson%20SC%5BAuthor%5D&cauthor=true&cauthor_uid=16106293), [Schofield C](https://www.ncbi.nlm.nih.gov/pubmed/?term=Schofield%20CJ%5BAuthor%5D&cauthor=true&cauthor_uid=16106293). Structural and mechanistic studies on anthocyanidin synthase catalysed oxidation of flavanone substrates: the effect of C-2 stereochemistry on product selectivity and mechanism. [Org Biomol Chem.](https://www.ncbi.nlm.nih.gov/pubmed/?term=The+Effect+of+C-2+Stereochemistry+on+Product+Selectivity+and+Mechanism) 2005;3(17):3117-26.
46. [Maugé C](https://www.ncbi.nlm.nih.gov/pubmed/?term=Maug%C3%A9%20C%5BAuthor%5D&cauthor=true&cauthor_uid=20138891), [Granier T](https://www.ncbi.nlm.nih.gov/pubmed/?term=Granier%20T%5BAuthor%5D&cauthor=true&cauthor_uid=20138891), [d'Estaintot B](https://www.ncbi.nlm.nih.gov/pubmed/?term=d'Estaintot%20BL%5BAuthor%5D&cauthor=true&cauthor_uid=20138891), [Gargouri M](https://www.ncbi.nlm.nih.gov/pubmed/?term=Gargouri%20M%5BAuthor%5D&cauthor=true&cauthor_uid=20138891), [Manigand C](https://www.ncbi.nlm.nih.gov/pubmed/?term=Manigand%20C%5BAuthor%5D&cauthor=true&cauthor_uid=20138891), [Schmitter J](https://www.ncbi.nlm.nih.gov/pubmed/?term=Schmitter%20JM%5BAuthor%5D&cauthor=true&cauthor_uid=20138891), et al. Crystal structure and catalytic mechanism of leucoanthocyanidin reductase from *Vitis vinifera*. [J Mol Biol.](https://www.ncbi.nlm.nih.gov/pubmed/?term=Crystal+structure+and+catalytic+mechanism+of+leucoanthocyanidin+reductase+from+Vitis+vinifera) 2010;397(4):1079-91.
47. [Levsh O](https://www.ncbi.nlm.nih.gov/pubmed/?term=Levsh%20O%5BAuthor%5D&cauthor=true&cauthor_uid=27805809), [Chiang Y](https://www.ncbi.nlm.nih.gov/pubmed/?term=Chiang%20YC%5BAuthor%5D&cauthor=true&cauthor_uid=27805809), [Tung C](https://www.ncbi.nlm.nih.gov/pubmed/?term=Tung%20CF%5BAuthor%5D&cauthor=true&cauthor_uid=27805809), [Noel J](https://www.ncbi.nlm.nih.gov/pubmed/?term=Noel%20JP%5BAuthor%5D&cauthor=true&cauthor_uid=27805809), [Wang Y](https://www.ncbi.nlm.nih.gov/pubmed/?term=Wang%20Y%5BAuthor%5D&cauthor=true&cauthor_uid=27805809), [Weng J](https://www.ncbi.nlm.nih.gov/pubmed/?term=Weng%20JK%5BAuthor%5D&cauthor=true&cauthor_uid=27805809). Dynamic conformational states dictate selectivity toward the native substrate in a substrate-permissive acyltransferase. [Biochemistry.](https://www.ncbi.nlm.nih.gov/pubmed/?term=Dynamic+Conformational+States+Dictate+Selectivity+toward+the+Native+Substrate+in+a+Substrate-Permissive+Acyltransferase) 2016;55(45):6314-6326.
48. [Hiromoto T](https://www.ncbi.nlm.nih.gov/pubmed/?term=Hiromoto%20T%5BAuthor%5D&cauthor=true&cauthor_uid=24121335), [Honjo E](https://www.ncbi.nlm.nih.gov/pubmed/?term=Honjo%20E%5BAuthor%5D&cauthor=true&cauthor_uid=24121335), [Tamada T](https://www.ncbi.nlm.nih.gov/pubmed/?term=Tamada%20T%5BAuthor%5D&cauthor=true&cauthor_uid=24121335), [Noda N](https://www.ncbi.nlm.nih.gov/pubmed/?term=Noda%20N%5BAuthor%5D&cauthor=true&cauthor_uid=24121335), [Kazuma K](https://www.ncbi.nlm.nih.gov/pubmed/?term=Kazuma%20K%5BAuthor%5D&cauthor=true&cauthor_uid=24121335), [Suzuki M](https://www.ncbi.nlm.nih.gov/pubmed/?term=Suzuki%20M%5BAuthor%5D&cauthor=true&cauthor_uid=24121335), [et](https://www.ncbi.nlm.nih.gov/pubmed/?term=Kuroki%20R%5BAuthor%5D&cauthor=true&cauthor_uid=24121335) al. Crystal structure of UDP-glucose: anthocyanidin 3-O-glucosyltransferase from *Clitoria ternatea*. [J Synchrotron Radiat.](https://www.ncbi.nlm.nih.gov/pubmed/24121335) 2013;20:894-8.
49. [Wilmouth R](https://www.ncbi.nlm.nih.gov/pubmed/?term=Wilmouth%20RC%5BAuthor%5D&cauthor=true&cauthor_uid=11796114), [Turnbull J](https://www.ncbi.nlm.nih.gov/pubmed/?term=Turnbull%20JJ%5BAuthor%5D&cauthor=true&cauthor_uid=11796114), [Welford R](https://www.ncbi.nlm.nih.gov/pubmed/?term=Welford%20RW%5BAuthor%5D&cauthor=true&cauthor_uid=11796114), [Clifton I](https://www.ncbi.nlm.nih.gov/pubmed/?term=Clifton%20IJ%5BAuthor%5D&cauthor=true&cauthor_uid=11796114), [Prescott A](https://www.ncbi.nlm.nih.gov/pubmed/?term=Prescott%20AG%5BAuthor%5D&cauthor=true&cauthor_uid=11796114), [Schofield C](https://www.ncbi.nlm.nih.gov/pubmed/?term=Schofield%20CJ%5BAuthor%5D&cauthor=true&cauthor_uid=11796114). Structure and mechanism of anthocyanidin synthase from *Arabidopsis thaliana*. [Structure.](https://www.ncbi.nlm.nih.gov/pubmed/11796114) 2002;10(1):93-103.
50. [Gonzalez E](https://www.ncbi.nlm.nih.gov/pubmed/?term=Gonzalez%20E%5BAuthor%5D&cauthor=true&cauthor_uid=29212707), [Johnson K](https://www.ncbi.nlm.nih.gov/pubmed/?term=Johnson%20KM%5BAuthor%5D&cauthor=true&cauthor_uid=29212707), [Pallan P](https://www.ncbi.nlm.nih.gov/pubmed/?term=Pallan%20PS%5BAuthor%5D&cauthor=true&cauthor_uid=29212707), [Phan T](https://www.ncbi.nlm.nih.gov/pubmed/?term=Phan%20TTN%5BAuthor%5D&cauthor=true&cauthor_uid=29212707), [Zhang W](https://www.ncbi.nlm.nih.gov/pubmed/?term=Zhang%20W%5BAuthor%5D&cauthor=true&cauthor_uid=29212707), [Lei L](https://www.ncbi.nlm.nih.gov/pubmed/?term=Lei%20L%5BAuthor%5D&cauthor=true&cauthor_uid=29212707), et al. Inherent steroid 17α, 20-lyase activity in defunct cytochrome P450 17A enzymes. [J Biol Chem.](https://www.ncbi.nlm.nih.gov/pubmed/?term=Inherent+steroid+17+alpha+%2C20-lyase+activity+in+defunct+cytochrome+P450+17A+enzymes.) 2018;293(2):541-556.
51. [Sansen S](https://www.ncbi.nlm.nih.gov/pubmed/?term=Sansen%20S%5BAuthor%5D&cauthor=true&cauthor_uid=17311915), [Yano J](https://www.ncbi.nlm.nih.gov/pubmed/?term=Yano%20JK%5BAuthor%5D&cauthor=true&cauthor_uid=17311915), [Reynald R](https://www.ncbi.nlm.nih.gov/pubmed/?term=Reynald%20RL%5BAuthor%5D&cauthor=true&cauthor_uid=17311915), [Schoch G](https://www.ncbi.nlm.nih.gov/pubmed/?term=Schoch%20GA%5BAuthor%5D&cauthor=true&cauthor_uid=17311915), [Griffin K](https://www.ncbi.nlm.nih.gov/pubmed/?term=Griffin%20KJ%5BAuthor%5D&cauthor=true&cauthor_uid=17311915), [Stout C](https://www.ncbi.nlm.nih.gov/pubmed/?term=Stout%20CD%5BAuthor%5D&cauthor=true&cauthor_uid=17311915), [et](https://www.ncbi.nlm.nih.gov/pubmed/?term=Johnson%20EF%5BAuthor%5D&cauthor=true&cauthor_uid=17311915) al. Adaptations for the oxidation of polycyclic aromatic hydrocarbons exhibited by the structure of human P450 1A2. [J Biol Chem.](https://www.ncbi.nlm.nih.gov/pubmed/?term=Adaptations+for+the+oxidation+of+polycyclic+aromatic+hydrocarbons+exhibited+by+the+structure+of+human+P450+1A2) 2007;282(19):14348-55.
52. [Wang A](https://www.ncbi.nlm.nih.gov/pubmed/?term=Wang%20A%5BAuthor%5D&cauthor=true&cauthor_uid=25555909), [Stout C](https://www.ncbi.nlm.nih.gov/pubmed/?term=Stout%20CD%5BAuthor%5D&cauthor=true&cauthor_uid=25555909), [Zhang Q](https://www.ncbi.nlm.nih.gov/pubmed/?term=Zhang%20Q%5BAuthor%5D&cauthor=true&cauthor_uid=25555909), [Johnson E](https://www.ncbi.nlm.nih.gov/pubmed/?term=Johnson%20EF%5BAuthor%5D&cauthor=true&cauthor_uid=25555909). Contributions of ionic interactions and protein dynamics to cytochrome P450 2D6 (CYP2D6) substrate and inhibitor binding. [J Biol Chem.](https://www.ncbi.nlm.nih.gov/pubmed/?term=Contributions+of+Ionic+Interactions+and+Protein+Dynamics+to+Cytochrome+P450+2D6+(CYP2D6)+Substrate+and+Inhibitor+Binding) 2015;290(8):5092-104.
53. Lygidakis A, Karuppiah V, Hoeven R, Ní Cheallaigh A, Leys D, Gardiner J, et al. Pinpointing a mechanistic switch between ketoreduction and "Ene" reduction in short-chain dehydrogenases/reductases. Angew Chem Int Ed Engl. 2016;55(33): 9596-600.
54. [Zhang Z](https://www.ncbi.nlm.nih.gov/pubmed/?term=Zhang%20Z%5BAuthor%5D&cauthor=true&cauthor_uid=15489165), [Ren J](https://www.ncbi.nlm.nih.gov/pubmed/?term=Ren%20JS%5BAuthor%5D&cauthor=true&cauthor_uid=15489165), [Clifton I](https://www.ncbi.nlm.nih.gov/pubmed/?term=Clifton%20IJ%5BAuthor%5D&cauthor=true&cauthor_uid=15489165), [Schofield C](https://www.ncbi.nlm.nih.gov/pubmed/?term=Schofield%20CJ%5BAuthor%5D&cauthor=true&cauthor_uid=15489165). Crystal structure and mechanistic implications of 1-aminocyclopropane-1-carboxylic acid oxidase (the ethyling forming enzyme). [Chem Biol.](https://www.ncbi.nlm.nih.gov/pubmed/?term=Crystal+Structure+and+Mechanistic+Implications+of+1-Aminocyclopropane-1-Carboxylic+Acid+Oxidase+(the+Ethyling+Forming+Enzyme)) 2004;11(10):1383-94.
55. Lindqvist Y, [Koskiniemi H](https://www.ncbi.nlm.nih.gov/pubmed/?term=Koskiniemi%20H%5BAuthor%5D&cauthor=true&cauthor_uid=19744497), [Jansson A](https://www.ncbi.nlm.nih.gov/pubmed/?term=Jansson%20A%5BAuthor%5D&cauthor=true&cauthor_uid=19744497), [Sandalova T](https://www.ncbi.nlm.nih.gov/pubmed/?term=Sandalova%20T%5BAuthor%5D&cauthor=true&cauthor_uid=19744497), [Schnell R](https://www.ncbi.nlm.nih.gov/pubmed/?term=Schnell%20R%5BAuthor%5D&cauthor=true&cauthor_uid=19744497), [Liu Z](https://www.ncbi.nlm.nih.gov/pubmed/?term=Liu%20Z%5BAuthor%5D&cauthor=true&cauthor_uid=19744497), et al., Structural basis for substrate recognition and specificity in aklavinone-11-hydroxylase from rhodomycin biosynthesis. [J Mol Biol.](https://www.ncbi.nlm.nih.gov/pubmed/?term=Structural+basis+for+substrate+recognition+and+specificity+in+aklavinone-11-hydroxylase+from+rhodomycin+biosynthesis) 2009;393(4):966-77.
56. **Figures**


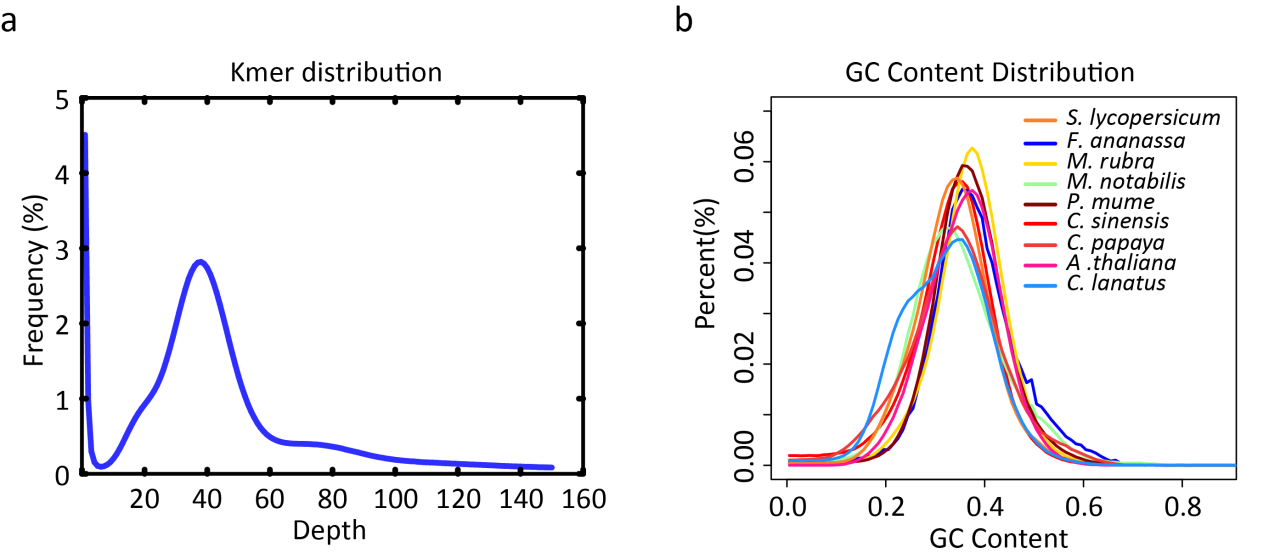


**Figure S1. Genome size evaluation and GC content distribution in *Myrica rubra*. (a)** Genome size evaluation from Kmer distribution. Distribution of the 17-kmer frequency based on 13.70 Gb of raw sequence data from 200-bp insert libraries. The depths of kmers (x-axis) are plotted against frequency (y-axis). The plot exhibits two peaks. The main peak represents the genome, and the smaller peak represents the repeats. The main peak depth is 37.56, and the *M. rubra* genome is estimated to comprise 304.38 Mb. **(b)** GC content distribution in the genomes of *M. rubra* and other species. The x-axis is the GC percentage, and the y-axis is the percentage of the genome. The other plant species, *Solanum lycopersicum*, *Fragaria* × *ananassa*, *M. rubra*, *Morus notabilis*, *Prunus mume*, *Citrus sinensis*, *Carica papaya*, *Arabidopsis thaliana*, and *Citrullus lanatus*, showed similar GC content distribution curves.


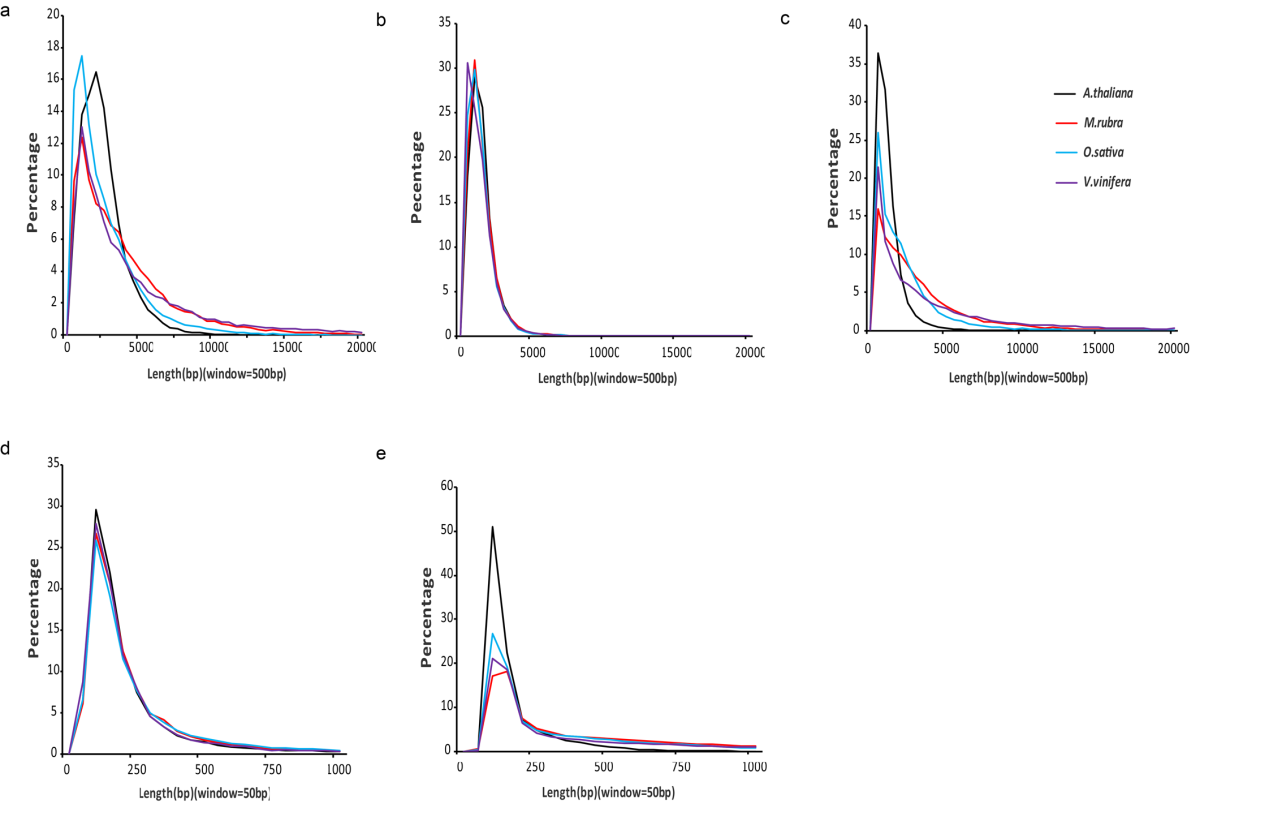


**Figure S2.** **Gene structure in *Myrica rubra*, *Arabidopsis thaliana*, *Oryza sativa*, and *Vitis vinifera*.** **(a)** Distribution of gene length. **(b)** Distribution of full coding sequence (CDS) length. **(c)** Distribution of single CDS length. **(d)** Distribution of full intron length. **(e)** Distribution of single intron length. Plots of *M. rubra* are similar to those of other species, indicating high accuracy of the predicted *M. rubra* gene model.


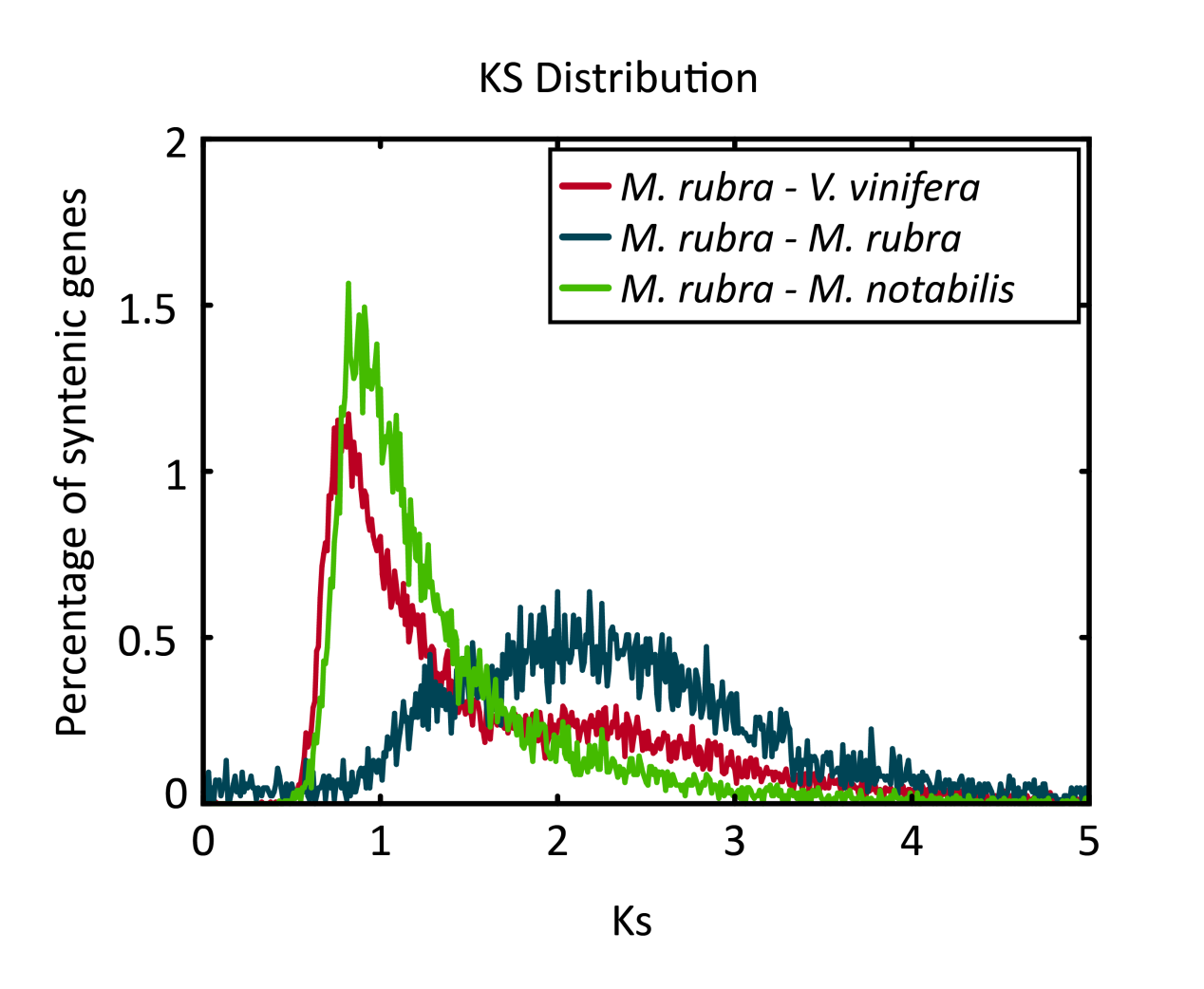


**Figure S3.** **Synonymous nucleotide substitution (Ks) distribution of homologous gene pairs in *Myrica rubra, Morus notabilis*, and *Vitis vinifera* comparisons.**


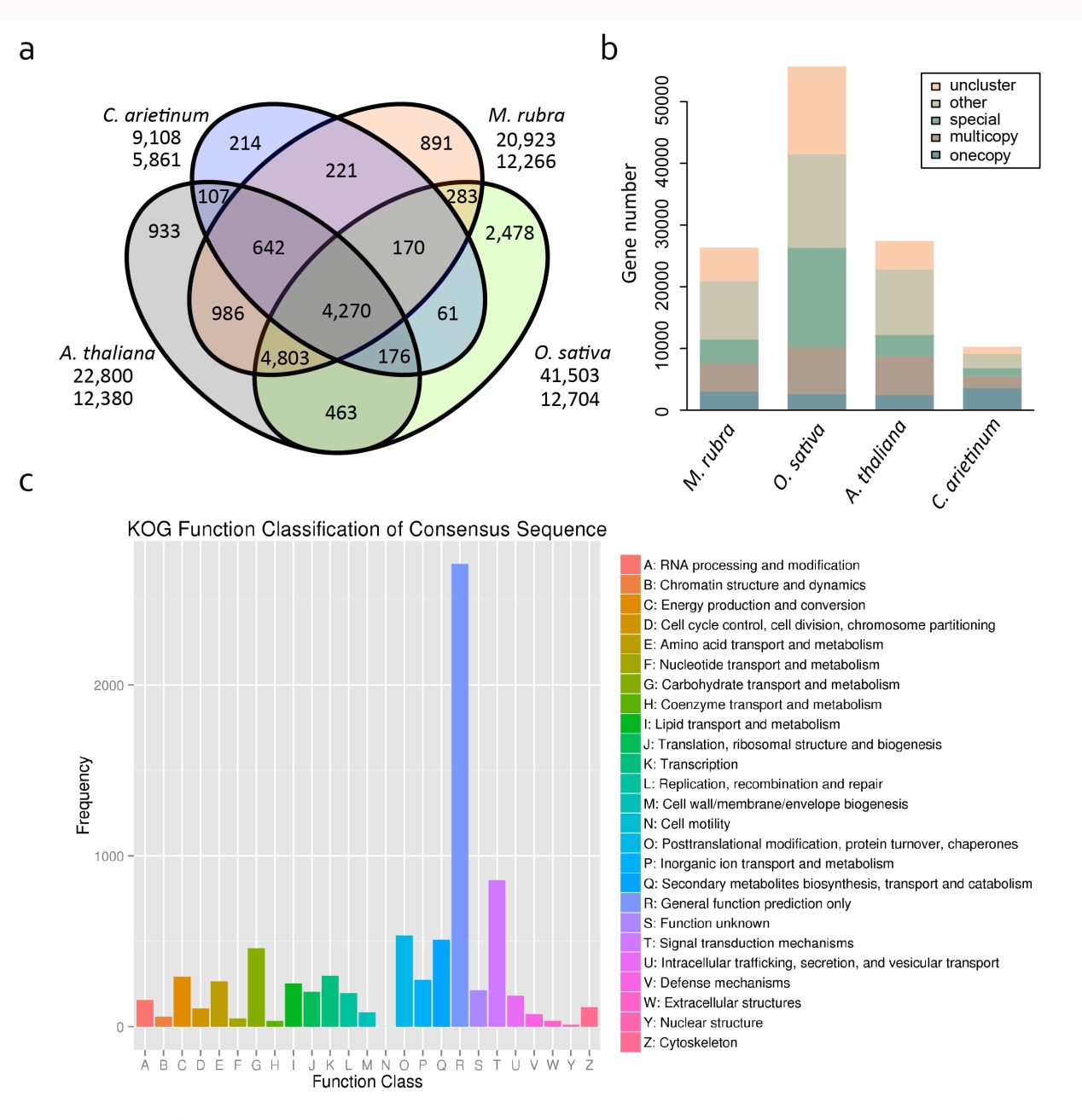


**Figure S4. Genome analysis of *Myrica rubra*, *Cicer arietinum*, *Arabidopsis thaliana*, and *Oryza sativa.* (a)** Clustering of gene families by OrthoMCL in *C. arietinum*, *A. thaliana*, *O. sativa*, and *M. rubra*. **(b)** Statistics of the genes involved in clustering. **(c)** Annotation and classification of genes unique to *M. rubra* based on KOG.


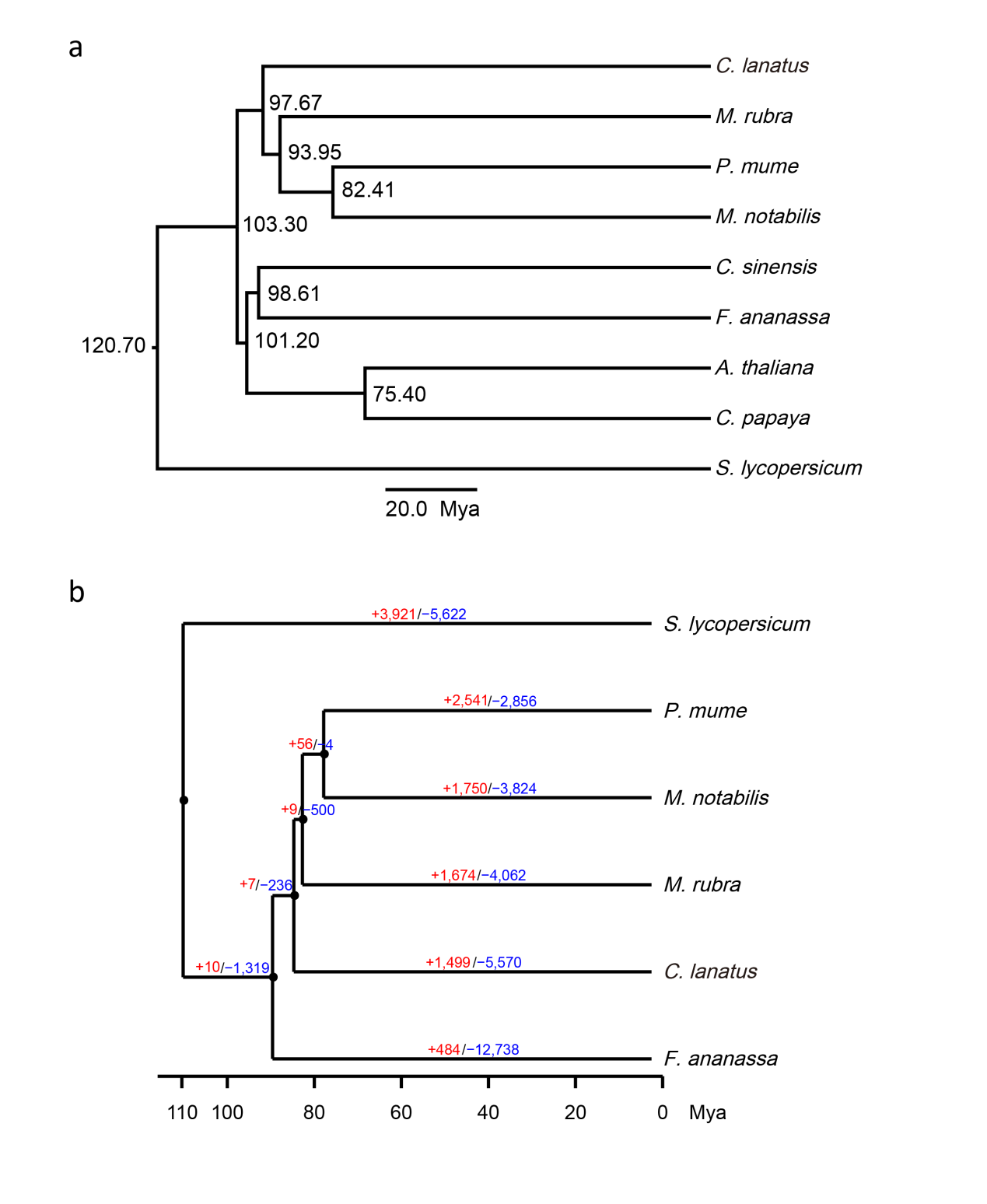


**Figure S5. *Myrica rubra* phylogenies based on single-copy genes common to other plants.** **(a)** *Myrica rubra* phylogeny inferred from 560 single-copy genes common to *Solanum lycopersicum*, *Prunus mume*, *Morus notabilis*, *Citrullus lanatus*, *Fragaria* × *ananassa*, *Citrus sinensis*, *Arabidopsis thaliana*, and *Carica papaya*. **(b)** *Myrica rubra* phylogeny inferred from 1,737 single-copy genes common to *S. lycopersicum*, *P. mume*, *M. notabilis*, *C. lanatus*, and *F.* × *ananassa*. Red and blue numbers respectively indicate expanded and contracted gene families. Mya, million years ago.


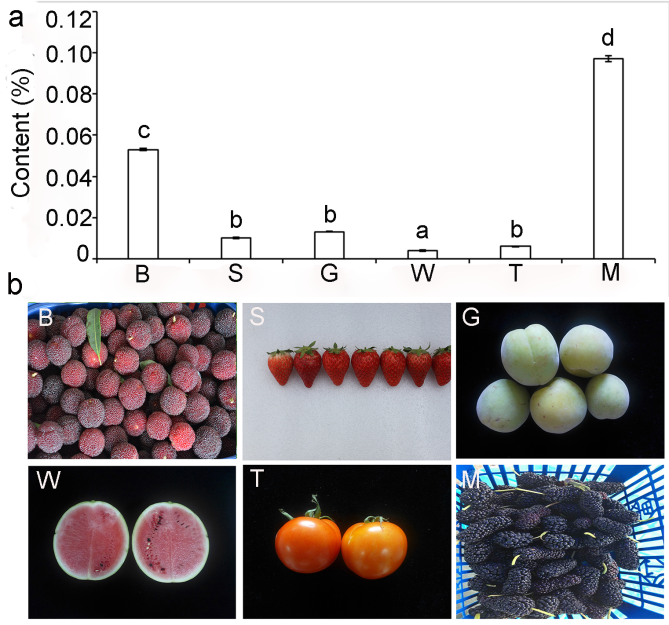


**Figure S6. Total flavones and colors of the six fruits compared in the present study.** **(a)** Total flavonoid content. **(b)** Fruit color: B, Chinese bayberry (*Myrica rubra*); S, strawberry (*Fragaria* × *ananassa*); G, green plum (*Prunus mume*); W, watermelon (*Citrullus lanatus*); T, tomato (*Solanum lycopersicum*); M, mulberry (*Morus notabilis*).


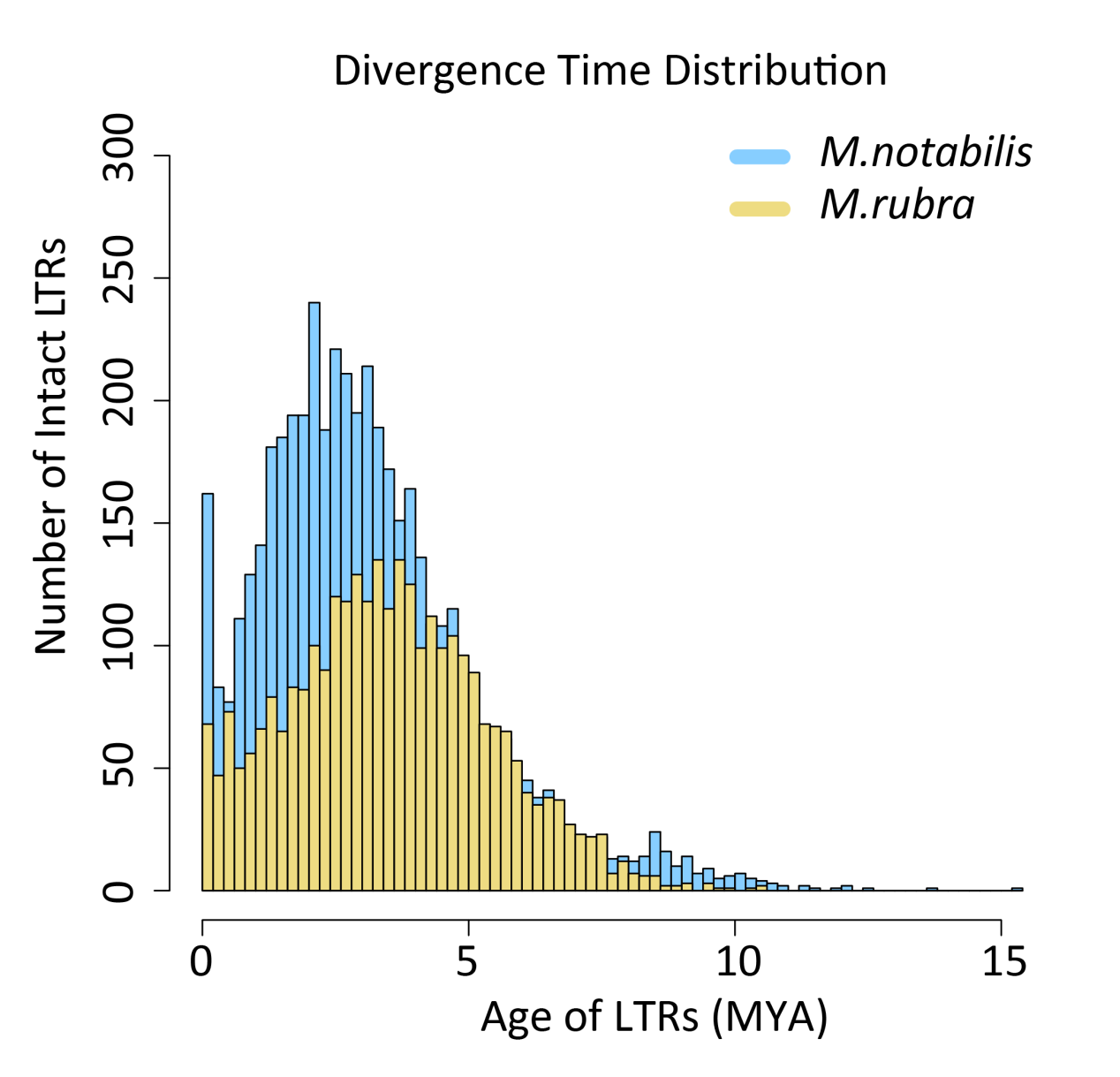


**Figure S7. Divergence distribution of sequence repeats in the *Myrica rubra* and *Morus notabilis* genomes.** Abundant long terminal repeat (LTR) insertions occurred recently in a narrow time window in the genomes of both species.
